# Factors affecting implementation of mindfulness in hospital settings: A qualitative meta-synthesis of healthcare professionals' experiences^✰^

**DOI:** 10.1016/j.ijnsa.2024.100192

**Published:** 2024-03-27

**Authors:** Randi Karkov Knudsen, Sine Skovbjerg, Elna Leth Pedersen, Camilla Littau Nielsen, Marie Højriis Storkholm, Connie Timmermann

**Affiliations:** a Department of Cardiology, Lillebaelt Hospital, Southern Denmark, Beriderbakken 4, Vejle 7100, Denmark; b Department of Gynecology and Obstetrics, Horsens Regional Hospital, Sundvej 30C, Horsens 8700, Denmark; c Centre for Research in Patient Communication, Odense University Hospital, Kløvervænget 12B, Odense C 5000, Denmark; d Department of Clinical Research, University of Southern Denmark, J.B. Winsløwsvej 19, Odense C 5000, Denmark; e Danish Center for Mindfulness, Department of Clinical Medicine, Aarhus University, Hack Kampmanns Plads 1-3, Aarhus C 8000, Denmark; f Hans Christian Andersen Children's Hospital, Odense University Hospital, Kløvervænget 23C, Odense C 5000, Denmark

**Keywords:** Healthcare professionals, Implementation, Meta-synthesis, Mindfulness, Qualitative research, Systematic Review

## Abstract

**Background:**

Researchers have found that mindfulness-based interventions can reduce stress and improve mental health in healthcare professionals, as well as support relationship building, communication, and compassionate care. However, few researchers have systematically examined what determines successful implementation in hospital settings, which is essential for integrating research in clinical practice.

**Objectives:**

The aim of this study was to synthesize qualitative data regarding healthcare professionals’ experiences of factors affecting implementation of mindfulness in hospital settings and outline recommendations for clinical practice.

**Design:**

A systematic review and meta-synthesis of qualitative studies.

**Data sources:**

A systematic search was conducted in six databases; Scopus, PubMed, CINAHL, PsycINFO (Ovid), Web of Science, and ProQuest Dissertations and Theses Global. The inclusion criteria were: 1) Healthcare professionals engaged in patient care in hospital settings, 2) Mindfulness-based interventions defined by Crane and colleagues’, and 3) Primary studies using a qualitative design.

**Review methods:**

Multiple researchers were engaged in screening, quality assessment, data extraction, and interpretation of the results. Thematic synthesis described by Thomas and Harden guided the data analysis. Reporting followed Enhancing Transparency in Reporting the Synthesis of Qualitative Research (ENTREQ).

**Results:**

Eighteen studies were included. We identified three overall themes of importance for successful implementation: 1) *Buying In*, 2) *Allocating time and space,* and 3) *Keeping it going*. The results revealed that cultural values, held beliefs about mindfulness, inter-professional relationships, and context-related factors such as time and space could affect implementation of mindfulness in hospital settings.

**Conclusion:**

Based on the results, we formulated eight recommendations to guide stakeholders and hospital management in planning implementation of mindfulness in hospital settings. However, to confirm the results, more research where mindfulness implementation is the primary aim is needed.

**What is already known about the topic**

• Exposure to high work-related pressure and emotionally challenging situations increases the risk of stress and burnout in healthcare professionals.
• Mindfulness-based interventions, such as Mindfulness-Based Stress Reduction and Mindfulness-Based Cognitive Therapy, are effective in improving mental wellbeing and reducing stress.
• There is a lack of qualitative evidence concerning successful implementation of mindfulness-based interventions in hospital settings.

Alt-text: Unlabelled box

**What this paper adds**

• Bringing mindfulness research into use in clinical practice requires careful attention to the implementation process.
• Important factors to consider when implementing mindfulness in hospital settings are cultural values, held beliefs about mindfulness, inter-professional relationships, and context-related factors, such as time and space for mindfulness training.
• Eight recommendations to guide the implementation process were formulated.

Alt-text: Unlabelled box

## 1 Introduction

There has been a growing interest in examining the effect of mindfulness training on healthcare professionals because of their exposure to high work-related pressure and emotionally challenging situations, which may ultimately increase the risk of stress and burnout (Garcia et al., 2019; Hall et al., 2016; Kriakous et al., 2021; Spinelli et al., 2019). A survey from 2021 showed that stress levels in healthcare professionals were 25.8 % higher than the general population (Couarraze et al., 2021). Researchers in other systematic reviews reported levels of burnout ranging from 30 to 60 % among healthcare professionals (Alanazy and Alruwaili, 2023; Claponea et al., 2022). This is not only damaging to their own health but may also lead to increased medical errors and negatively affect patient safety (Garcia et al., 2019; Hall et al., 2016; Hodkinson et al., 2022). Therefore, it is relevant to invest in effective interventions to prevent stress and burnout and improve wellbeing and thereby enhance the quality of patient care.

Mindfulness-based interventions support present moment awareness and the development of greater attentional, emotional, and behavioral self-regulation (Crane et al., 2017). Researchers who published a meta-analysis from 2021 found that mindfulness-based interventions were among the most effective interventions in improving mental wellbeing compared to other types of mental health promoting interventions (van Agteren et al., 2021). In occupational settings, including healthcare, a number of systematic reviews and meta-analyses have shown that mindfulness-based interventions reduce symptoms of stress, anxiety, and depression (Kriakous et al., 2021, Spinelli et al., 2019, Vonderlin et al., 2020) and increase wellbeing, compassion, mindfulness, and job-satisfaction (Kriakous et al., 2021; Vonderlin et al., 2020). Most researchers who have investigated mindfulness-based interventions have applied quantitative designs, measuring the effect of the intervention, but some qualitative reviews have also been conducted (DeMauro et al., 2019; Hunter, 2016; Morgan et al., 2015; Wu et al., 2021). In these reviews, researchers found that healthcare professionals' experience was a positive link between mindfulness training and the ability to practice self-care, manage stress in the work environment, and develop agency in their work life (DeMauro et al., 2019; Hunter, 2016; Morgan et al., 2015; Wu et al., 2021). In addition, healthcare professionals described that mindfulness training enhanced their ability to regulate emotions, listen, be present, and practice compassionate patient care (DeMauro et al., 2019; Hunter, 2016; Morgan et al., 2015; Wu et al., 2021). As such, the evidence base documenting a beneficial effect of mindfulness training on healthcare professionals' mental health and clinical practice is promising. However, Micklitz et al. (2021) conducted a realist review about how and why workplace mindfulness-based interventions work or do not work. The reviewers concluded that mindfulness training helped to build resources to manage stress and enhance wellbeing, but a supportive environment was necessary for the benefits to be reaped (Micklitz et al., 2021). If this is not the case, employees might prefer to protect current practice instead of engaging in new routines; i.e. mindfulness (Micklitz et al., 2021). This is supported by existing implementation research, from which investigators have suggested that rigorous evidence is rarely enough to guarantee uptake into clinical practice (Harvey and Kitson, 2015). The integrated Promoting Action on Research Implementation in Health Services (i-PARIHS) framework describes successful implementation as a complex process that can be defined as a function of the evidence of the innovation being implemented, the recipients who are affected by the implementation, the qualities of the context, and the facilitation process; i.e., how the implementation is being enacted (Harvey and Kitson, 2016).

To our knowledge, no qualitative systematic review has aimed to identify factors of relevance to secure a successful implementation of mindfulness-based interventions in hospital settings. Thus, the aim of this study was first to synthesize the qualitative data regarding healthcare professionals' experiences of facilitating and inhibiting factors affecting implementation of mindfulness-based interventions in hospital settings, and second to outline recommendations to inform clinical practice based on the findings.

## 2 Methods

While quantitative research provides important knowledge of the effectiveness of interventions, qualitative research is suitable for increasing the understanding of factors related to implementing interventions and overcoming barriers to the use of new knowledge (Thomas and Harden, 2008). Qualitative research is underpinned by the interpretive paradigm, which values subjective perspectives of individuals and seek to understand meaning in the social context (Pearson et al., 2011). Meta-synthesis is a structured method for systematic reviewing and synthesizing finding from qualitative research (Korhonen et al., 2013). In this meta-synthesis, we used thematic synthesis guided by Thomas and Harden (2008). This approach is suitable to address questions about factors affecting intervention implementation from the point of view of the people involved. Bringing together findings about these factors from primary qualitative research can provide important knowledge to guide decision-making in relation to mindfulness implementation (Thomas and Harden, 2008). The research process consisted of the following steps: 1) Identifying the research question, 2) Locating relevant studies through a systematic search, 3) Screening, selecting, and critically assessing the articles, 4) Data extraction, 5) Data analysis and presentation of a qualitative synthesis, which go beyond the content of the original studies. The purpose was to produce knowledge intended to inform clinical practice (Thomas and Harden, 2008).

A protocol was registered in PROSPERO on 7 April 2023 (ID CRD42023411192), and a small adjustment was made on 6 June 2023 after the initial literature search. This involved adding a frame to define what counted as mindfulness-based interventions to ensure inclusion of knowledge based on the strongest evidence (Crane et al., 2017). The reporting was guided by the Enhancing Transparency in Reporting the Synthesis of Qualitative Research (ENTREQ) (Tong et al., 2012), Supplementary Material File 1.

### 2.1 Search strategy

A comprehensive search strategy was developed in collaboration with an experienced research librarian. We used the search tool 1) Population, 2) Exposure, and 3) Outcome (Moola et al., 2015). We searched six databases appropriate for identifying studies in healthcare: Scopus, PubMed, CINAHL, PsycINFO (Ovid), Web of Science, and ProQuest Dissertations and Theses Global, using free text with proximity operators. As an example, Table 1 illustrates the search strategy performed in Scopus. The search strategy was modified to fit each database using relevant Mesh terms and Subject Headings in CINAHL, PubMed, and PsycINFO. To optimize the search strategy for locating qualitative research, we followed Roger's recommendations and added a search filter in PsycINFO and PubMed: (Interview*.af. Or experience*).af. OR qualitative.tw. (Rogers et al., 2018). No date or language limit was applied.

**Table 1:** Search strategy in Scopus

	**Keywords**
Population	(TITLE-ABS-KEY ((("healthcare OR "health care") W/2 (worker* OR professional* OR employee* OR personnel"))) OR TITLE-ABS-KEY ("hospital staff" OR "health personnel" OR nurs* OR midwi* OR physician* OR clinician* OR doctor*)
**AND**	
Exposure	TITLE-ABS-KEY (Mindful* OR meditation OR mbsr OR mbi)
**AND**	
Outcome	TITLE-ABS-KEY (Qualitative OR interview* OR "focus group" OR "focus groups" OR experience* OR ethno*)

The literature search was performed between 20 March 2023 and 25 March 2023 and repeated on 26 June 2023. Supplementary Material File 2 provides a full list of searches from 26 June 2023. A reference and citation search of the included studies and other qualitative meta-synthesis concerning mindfulness training to healthcare professionals (DeMauro et al., 2019; Hunter, 2016; Micklitz et al., 2021; Morgan et al., 2015; Wu et al., 2021) was performed in Web of Science and Scopus on 28 June 2023.

### 2.2 Inclusion and exclusion criteria

The inclusion criteria were the following.

1) Healthcare professionals engaged in patient care and working in hospice, mental, or somatic hospital settings, who had attended a mindfulness-based intervention.
2) Mindfulness-based interventions was defined by the framework described by Crane and colleagues’ (Crane et al., 2017). This framework is based on first generation mindfulness-based interventions; i.e., Mindfulness-Based Stress Reduction, Mindfulness-Based Cognitive Therapy, and a range of programs developed from these. In this definition, the program must be informed by contemplative theories, support present moment focus, and the development of greater attentional, emotional, and behavioral self-regulation. Mindfulness-based interventions require a qualified teacher who guides systematic training in formal and informal mindfulness meditation practice through an inquiry-based learning process. The program structure, length, and delivery form can be adjusted to fit the population and context (Crane et al., 2017).
3) Primary studies using a qualitative design, with focus group or individual interviews for data collection, describing healthcare professionals’ perspectives of factors affecting implementation of mindfulness in hospital settings. Exclusion criteria were studies where the mindfulness-based intervention was based solely on apps, self-help books, or online programs without a teacher, and studies including only brief quotations from surveys.

### 2.3 Screening the search results

**Fig. 1:**
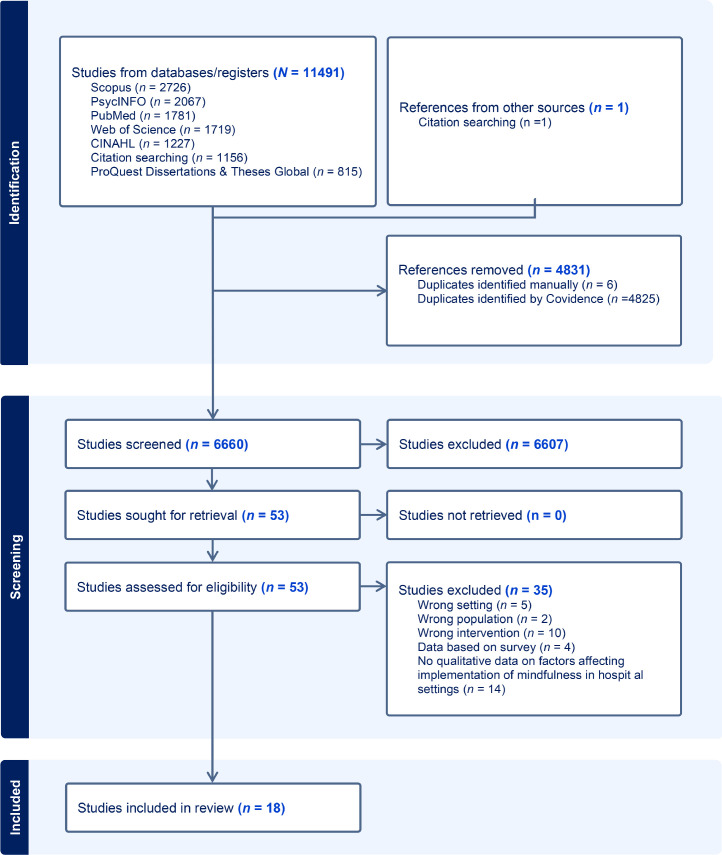
PRISMA flowchart, *N =* total number, *N* = subgroup number.

All identified studies were uploaded to Covidence, an online software tool designed to streamline the systematic review process (Covidence.org). Duplicates were removed, and the search results were screened by title, abstract, and full text by independent researchers. The first author, RKK, screened all the studies, and CLN, ELP, and CT independently screened one third of the studies at each stage. This resulted in some ambiguities concerning the definition of mindfulness-based interventions, which led to the use of Crane and colleagues' framework for clarification (Crane et al., 2017). Disagreement in the screening process was discussed and resolved among the researchers, and uncertainty concerning whether the intervention could qualify as a mindfulness-based intervention was clarified by an additional researcher (SS) with expertise in mindfulness research. Reasons for excluding full-text screened studies were documented in the PRISMA flowchart illustrating the search results and selection process (Fig. 1). A full list of the excluded studies and description of the reason for exclusion is shown in Supplementary Material File 3.

### 2.4 Quality assessment

The trustworthiness of the results depends on the quality of the included studies (Korhonen et al., 2013). Therefore, we used the Critical Appraisal Skills Program for qualitative studies (CASP) for quality assessment and transparency. The tool contains 10 questions, including prompts or sub-questions to consider under each question. The questions can be answered with a yes, no, or unclear (Critical Appraisal Skills Programme, 2018). We used the scoring system described by Butler et al. (2016), where 0 points are allocated for no, 1 for yes, and 0.5 for unclear or partly (Butler et al., 2016). We used the term partly if some but not all sub-questions could be answered yes. Table 2 illustrates the score of each included study. Quality assessment of each study was performed by two independent researchers. RKK assessed all the studies, and CLN, ELP, and CT assessed one-third each. There were few ambiguities, but these were resolved through a group discussion. Most of the studies were of high quality, and all contributed to knowledge about factors affecting mindfulness implementation in hospital settings. Thus, to use all relevant information and describe the current level of evidence, we did not exclude any studies.

**Table 2:** CASP (Critical Appraisal Skills Program) quality assessment.

1. Was there a clear statement of the aims of the research?

2. Is a qualitative methodology appropriate?

3. Was the research design appropriate to address the aims of the research?

4. Was the recruitment strategy appropriate to the aims of the research?

5. Was the data collected in a way that addressed the research issue?

6. Has the relationship with the researcher and participants been adequately considered?

7. Have ethical issues been taken into consideration?

8. Was the data analysis sufficiently rigorous?

9. Is there a clear statement of the findings?

10. How valuable is the research?

### 2.5 Data extraction and synthesis

Two researchers independently assessed and extracted descriptive data about aim, population, intervention, methods (Table 3) and content from the Results and Discussion sections involving facilitating and inhibiting factors affecting the implementation of mindfulness in hospital settings. Again, RKK extracted data from all the studies, and CLN, ELP, and CT extracted data from one-third each. Supplementary Material File 4 illustrates the reviewer guide for this process. The included studies were uploaded to the software program Nvivo, version 14 (QRS International) and analyzed using thematic synthesis (Thomas and Harden, 2008). The research question – “*which facilitating and inhibiting factors can be identified as relevant for implementation of mindfulness in hospital settings, based on healthcare professionals’ experiences”* guided the analysis in an inductive process, where the codes and themes were identified based on data from the primary studies (31). The analysis was performed in three stages: 1) Coding text, 2) Developing descriptive themes, and 3) Generating analytical themes to produce higher-order themes that go beyond the findings from the primary studies (Thomas and Harden, 2008). The first author (RKK) and last author (CT) discussed possible codes to use in a line-by-line coding process of the extracted data, and afterwards, the first author (RKK) performed the coding. Thirteen codes were created and subsequently organized into six preliminary descriptive themes based on similarities of the codes. Five members of the research team (RKK, SS, CLN, ELP, and CT) discussed the data coding and preliminary themes. This led to further refinement of the themes and the generation of three analytical themes describing facilitating and inhibiting factors of importance for implementation of mindfulness in hospital settings. The analysis process with examples of extracted data, codes, preliminary themes, and analytical themes can be found in Supplementary Material File 5.

**Table 3:** Included studies. MBI= Mindfulness-based intervention, MBSR= Mindfulness-Based Stress Reduction, MBCT= Mindfulness-Based Cognitive Therapy, MSC= Mindful Self-Compassion, RCT=Randomized clinical trial, AIDS= Acquired immunodeficiency syndrome, USA= United States of America, UK= United Kingdom.

First author, year of publication, country	Aim of the study	Participants/ speciality/ setting	Type of intervention	Design/qualitative data collection method	Method for data analysis	Short description of main results
Minichiello et al. (2020) USA	To explore residents’ experiences and personal application of how a 10-hour multispecialty mindfulness training course (STREaM) changed their thoughts and behaviours during clinical work.	**Population**: 36 residents **Speciality**: family medicine, ophthalmology, paediatrics, and anaesthesiology. **Experience**: first year residents. Sex: no info. **Age**: no info.	**MBI**: STREaM is a 10-hour mindfulness-based skills course created for first-year residents. Sessions varied between 60 and 90 min. Included meditation, self-reflection, dyad conversations, and group dialogue. **Home practice**: brief mindfulness practices between the sessions. **Location**: At the university. **Instructors**: A mindfulness program manager with 24 years of experience with training and teaching mindfulness, a paediatric hospitalist, and a family physician, both trained in MBSR.	Qualitative design. 36 semi-structured individual phone interviews, using a facilitator guide, within 1 month of completing the MBI.	Qualitative data analysis, type not specified. Reviewers independently coded the transcripts and dialogued to reach consensus around themes.	All residents acknowledged the potential usefulness of mindfulness training during residency. Four themes related to clinical application of mindfulness training were identified - integrating brief moments of mindfulness, self-awareness, relational presence with patients and maintaining perspective during clinical encounters and residency training. (p. 356)
Brun et al. (2023) Spain	To assess healthcare professionals’ experiences of the impact of a compassion-centred mindfulness program on work practice.	**Population**: 3 nurses, 6 physicians, 1 social worker, 1 nurse manager. **Speciality**: no info. **Experience**: > 10 years of practice experience Sex: 91 % female. **Age**: mean age 43,55.	**MBI**: MB CARE program, adopted from MBSR and MSC, full day sessions 4 days in a row (*n* = 4) or once a week for 4 weeks (*n* = 7). Included meditation from MBSR, communication training and dialog with the frame from MBSR. **Home practice**: no info. **Location**: At the hospital (*n* = 7) or at a yoga/meditation centre (*n* = 4). **Instructor**: experienced MBSR/compassion teacher.	Qualitative design. 11 Semi-structured individual phone interviews, 1–2 years after completing the MBI.	King's template analysis method	The program had an overall positive impact on healthcare professionals’ ability to feel compassion and kindness toward their patients and themselves, enhance their attention to their patient´s needs and they became better at accepting the difficult work experiences or those their patients experienced. They saw the programs as personal development with indirect effects on work (p. 1).
Weisbaum (2021) Canada	To explore how physicians experience, make sense of, and engage with a five-week Applied Mindfulness program and the impact on their personal wellbeing in the context of their daily lives.	**Population:** 45 physicians. **Speciality:** Cardiology, emergency, surgery, neurology, psychiatry, oncology etc. **Experience: a**verage 14 years of practice experiences. Sex**:** no info. **Age**: average age 43.	**MBI:** Applied Mindfulness program based on teachings of Thich Nhât Hanh, 5 sessions, 2 h per week, and a booster session 3 months after the program. **Home practice**: minimum one mindfulness practice between sessions. **Location**: At the hospital. **Instructor**: Two mindfulness teachers with extensive experiences. Trained with Thich Nhat Hanh and Plum Village.	Qualitative design. 28 semi-structured interviews within 4 weeks of completing the MBI, in-sessions participant observation and focus groups conducted 16 months after completing the MBI.	Thematic analysis	Participants developed a foundational competency in Applied Mindfulness that benefitted their personal sense of wellbeing and enhanced their interaction with patients, colleagues, and administrators. Key themes: Initial intention, Establishing a mindful environment, Development and application of practices and concepts, Broadening the conceptualization of mindfulness and Mindfulness becomes a way of life (p. ⅲ).
Aeschbach et al. (2021) Germany	To explore resident physicians’ experiences of and perceived effects of a tailored mindfulness-based intervention and secondary to compare the effects with a control group that included text-based learning of mindfulness.	**Population:** 21 resident physicians from an intervention group and 14 from a control group **Speciality: w**orking in a wide range of medical disciplines in major hospital (information from protocol) **Experience:** mean 3,3 years of **p**ractice experience Sex**:** 66,7 % female in the intervention group, 69,2 % female in the control group. **Age:** mean age 30,7.	**MBI**: Adapted MBSR, 8 sessions, 2.5 h per week, plus 1-day 6-hour silent retreat. Focus on tailoring mindfulness to resident specific topics. The control group read the written material from the course. **Home practice:** No info. **Location**: No info. **Instructor**: physician and certified MBSR or MBCT teacher (information from protocol)	A qualitative study as a part of a larger RCT. 35 Individual interviews, using a semi-structured guide,1–6 month after completing the MBI.	Thematic analysis guided by Brun and Clarke	Physicians reported that the program helped them to integrate mindfulness into everyday life, increased their self-awareness, equanimity and wellbeing and had positive effects on self-care and interaction with patients. The control group perceived minor effects (p. 1).
Knudsen et al. (2021) Denmark	To explore physicians' and nurses' experiences of how attending a MBSR-course influenced their clinical practice and interaction with colleagues and patients	**Population:** 3 physicians, and 3 nurses. **Speciality:** Cardiology. **Experience:** Average 5,6 (1–17) years of practice experience. Sex**:** 100 % female. **Age** Average age 32,6.	**MBI**: MBSR, 8 sessions, 2. h per week, plus 1-day (6 h) silent retreat. **Home practice**: 45–60 min. mindfulness practice daily between sessions. **Location**: at the hospital. I**nstructor**: Certified MBSR teacher.	Qualitative design. 6 individual interviews, 2–3 weeks after completing the MBI.	Interpretive phenomenological analysis guided by Smith	Healthcare professionals reported that the MBSR course changed their ability to focus, prioritize and stay calm in busy situations. They described an increased acceptance of their own limitations, better understanding of colleagues and greater awareness of the unique patient (p. 1).
Negus and Grobler (2021) South Africa	To explore healthcare professionals understanding of mindfulness following a training course, their degree of confidence in sharing the skills they learned with colleagues and patients and benefits gained, challenges faced, and changes noted	**Population:** 10 nurses, 1 psychiatrist, 2 psychiatrists in training, 2 social workers **Speciality:** Psychiatric **Experience: n**o info. Sex**: n**o info. **Age:** no info.	**MBI**: Adapted MBSR, 12 sessions, 1 h twice a week for 6 weeks. **Home practice**: mindfulness practice between sessions. **Location**: At the psychiatric hospital. I**nstructor**: Two psychiatrists, with experience with mindfulness through self-study, and self-practice using resources from mindfulness experts (Jon Kabat-Zinn, Jack Cornfield and Bruno Cayoun).	Qualitative design. case study with 15 semi-structured individual interviews, 4–6 weeks after completing the MBI.	Thematic analysis	Participants perceived that the understanding of mindfulness expanded with practice, they had unexpected experiences during the mindfulness course, such as increased awareness and understanding of self, others, and the environment, understanding the need for self-care, improved self-control and communication, but also challenges with lack of structure, time, consistency and unpleasant emotions (p. 1–7).
Lebares et al. (2020) USA	To identify critical barriers and enablers of adopting mindfulness-based cognitive interventions for surgical trainees, delivered and studied on three separate occasions.	**Population:** 64 physician trainees in the intervention group, 37 in the control group, 14 program directors, 7 administrators, 8 senior residents. **Speciality:** surgery, family practice, obstetrics- and gynaecology. **Experience:** training level: PGY 1–5 s. Sex**:** 36 % female in study 1, 65 % female in study 2, 50 % female in study 3 **Age:** No info.	**MBI:** Enhanced Stress Resilience Training, adopted from MBSR, Study 1: 8 sessions, 2 h per week and a hike retreat. Study 2: 6 sessions, 1.5 h per week, hike retreat. Study 3: 6 sessions, 1.5 h per week, hike retreat. **Home practice**: 20 min of mindfulness practice daily. **Location**: UCSF campus. I**nstructor**: certified MBSR teacher.	Mixed method from 3 studies. 2 RCT, 1 cohort study. Qualitative data consist of interviews with program directors and administrators before and after the intervention (*n* = 21), focus group with senior residents’ pre-intervention (*n* = 8), and focus group with physician trainees post intervention (*n* = 64). Data were collected through 3 years.	Grounded theory analysis - using both inductive a deductive coding. Consolidated Framework for Implementation Research guided the process.	The culture surrounding the intervention, infrastructure supporting the intervention, and adaptability of the intervention embodied the most influential factors involved in implementing the enhanced stress resilience training in the departments (p. 328).
Muir and Keim-Malpass (2020) USA	To assess the feasibility of a MBI for emergency nurses and patient care technicians, to investigate changes in burnout scores before and after the intervention, and to understand clinician perspectives about drivers to burnout.	**Population:** 26 nurses and 9 patient care technicians **Speciality:** emergency department level 1 trauma centre. **Experience:** mean 2,3 years of practice experience. Sex**:** 77,14 % female. **Age: m**ean age 31,63.	**MBI**: Adopted MBSR, 3 sessions, 1.5 h once a month during staff meetings, in-person, through videoconferencing live or after the live sessions in a recorded version. Drop-in meditation (on a non-scheduled basis) in the first hour of the workday shift in no occupied trauma bays in the department **Home practice**: minimum 2 mindfulness meditations per week between the sessions. **Location**: At the hospital. **Instructor**: certified MBSR teacher.	Mixed methods. 5 individual in-person semi-structured interviews, 13–16 months after the completing the MBI.	Thematic analysis	The findings revealed benefits of the mindfulness training on clinician resiliency and opportunities to address burnout. Five themes were identified: prioritization distress, change fatigue, self-protection through superficiality, intentional response, and community amid chaos (p. 205).
Nissim et al. (2019) Canada	To understand experiences of oncology healthcare providers who participated in a MBI, how it affected them and how it could be optimized.	**Population:** 10 oncology healthcare providers (OHP)- (nurses, oncologist and other OHPs). **Speciality: o**ncology **Experience:** no info. Sex**:** no info. **Age:** no info.	**MBI:** Compassion, Presence and Resilience Training, 8 sessions,1.5 h per week. The program included a range of formal and informal mindfulness practices and facilitator-led discussion informed by Kabat-Zinn, Segal, Epstein, and Neff. **Home practice:** no info. **Location**: At the hospital. **Instructor:** Psychiatrist with extensive experiences in mindfulness training.	Qualitative design. 10 semi-structured interview, 1–5 months after completing the MBI.	Thematic analysis, guided by Patton's utilization-focused qualitative evaluation approach.	The participant identified benefits in – learning to pause, acquiring a working definition of stress and self-care, becoming fully present, building self-compassion, receiving organizational acknowledgment, and recognition of stress. Participant-Identified Challenges were sharing vulnerability within inter-professional teams and committing to a sitting meditation (p. 30).
Pan et al. (2019) China	To evaluate a mindfulness-based intervention for nurses providing care for people living with HIV.	**Population:** 20 nurses **Speciality:** AIDS department **Experience:** Average 6,84 (2–23) years of practice experience. Sex**:** 94,7 % female. **Age: m**ean age 27,28 (21–41).	**MBI**: Mindful living with stress (MLWS), based on considerations of the MBSR and MAP protocol. 6 sessions, 2 h once a week for 6 weeks. Include mindful meditation, body scan, and yoga. **Home practices:** daily mindfulness practices between sessions. **Location**: a private, quiet room. **Instructor**: the principal investigator facilitated the intervention. No info. of qualifications.	Mixed methods. Qualitative components consist of 20 in-depth interviews conducted by a research assistant, using a semi-structured interview guide. Conducted post-intervention.	Thematic analysis. (By two researchers)	Nurses experienced a decrease in work and daily life pressures, improvements in communications with patients, colleagues, and families, better regulation of negative emotions and acceptance of other people and attention (p. 3131).
Hunter et al. (2018) UK	To explore how midwives who attended a mindfulness course perceived that it impacted their professional practice	**Population:** 9 midwives. **Speciality:** large maternity trust. **Experience: p**ractice experience ranged from newly qualified to experienced midwife. Sex**:** 100 % female. **Age:** no info…	**MBI:** Adapted version of MBCT, 8 sessions, 1–1.5 h per week for 8 weeks. **Home practice**: no info. **Location**: At the maternity trust. **Instructor:** no info.	Qualitative design. 9 semi-structured individual interviews. Conducted post-intervention.	Interpretive phenomenological analysis guided by Smith	While some participants initially found aspects of the course challenging, those that committed to the concept gained an increased awareness of self and of the self as a situated entity which gave them a sense of control, and enabled them to reconnect with themselves, their colleagues, work, and the women they cared for and to face the future with confidence and positivity (p. 1236).
Slatyer et al. (2018) Australia	To explore the acceptability, feasibility, and applicability of a brief mindful self-care program	**Population:** 16 nurses. **Speciality: a**cute care. **Experience:** 81 % had > 20 years of practice experiences. Sex**:** 100 % female. **Age:** 87,5 % were > 40 years, 12,5 % were < 40 years.	**MBI:** MCSR (mindful self-care resiliency program) adapted from MBCT, total of 12 h, 1-day workshop followed by 4 weekly sessions, 1.5 per hour per week. **Home practice:** 10–20 min of mindfulness practice per day between sessions. **Location:** a private room at the hospital. I**nstructor**: registered clinical psychologist, with more than 6 years of experience in delivering mindfulness-based training to health professionals.	Mixed methods. Qualitative components consist of 16 unstructured individual interviews, 5 via phone and 11 in person, after completing the MBI.	Thematic analysis, guided by Braun and Clarke	The nurses’ experienced that they gained perspective and insight to their own stress responses, developed feelings of inner calm, and taking time to self-care after attending the program. The program was found to be feasible and acceptable for nurses working in an acute care hospital (p. 610).
Mealer et al. (2017) USA	To gather information for the adaptation of an MBCT resilience intervention.	**Population:** 33 nurses. **Speciality:** critical care. **Experience:** no info Sex**:** 96% female. **Age:** no info.	**MBI**: MBCT, 8 sessions, 2 h per week. **Home practice:** 30–60 min daily 6 days a week. **Location**: **Instructor**: qualified MBCT-teacher.	Qualitative design. 11 focus group with 33 nurses, were conducted by videoconference.	Manual qualitative analysis	The participant identified potential barriers to adherence, incentives for adherence, preferred qualifications of instructors, didactic content, and intensive care unit-specific issues to be addressed (p. 359).
Byron et al. (2015) USA	To explore staff and leaders’ perceptions of the implementation of mindfulness training in adolescent mental health units, including barriers, benefits and the organizational culture and context.	**Population:** 3 organizational leaders, 15 unit managers and direct care staff (psychiatrists, nurses, social workers, psychologist and occupational therapists) **Speciality:** mental health, Adolescent inpatient unit. **Experience:** no info. Sex**:** no info. **Age:** no info.	**MBI**: Adapted MBSR, 8 sessions, 2 h per week. **Home practice**: 45 min of mindfulness practice daily 6 days a week. **Location**: at the hospital. **Instructo**r: qualified mindfulness specialist	Qualitative design. 3 semi-structured focus groups and 1 by request one-on-one interview. 1 focus group were with leaders, and 2 were with inpatient teams. The interviews were conducted a year after completing the MBI.	Grounded theory analysis - using an inductive approach for coding the data, guided by Bradley	Facilitating factors to implementing mindfulness in the department included organizational leadership at several levels, securing initial buy-in, attention to logistical factors including scheduling and location, past experience with mindfulness, identification of local champions, and an acculturative process of attraction. Barriers was insufficient time and coverage to allow direct care providers to participate, without using personal time and insufficient preparation for the new initiative (p. 861).
Lyddy et al. (2016) USA	To explore how healthcare professionals use and perceive mindfulness practices at work.	**Population:** 25 healthcare professionals (4 disaster recovery worker, 4 physicians, 2 nurses, 5 Chaplain, 5 administrators, 3 analysts, 2 social workers) **Speciality:** not specified. **Experience:** no info. Sex**:** 72 % female. **Age:** 64 % < 40, 36 %>40 years.	**MBI**: 1 or 16-hour adaptations of the "Joy of Living program", including long mindfulness training and brief meditation practices to facilitate integration to job activities. Differ from MBSR by not including yoga or retreat components and the training occurred over various time frames to accommodate work schedules. **Home practice**: no info. **Location**: no info. **Instructor**: authorized to lead the Joy of Living program.	Qualitative design. Focus group interview using a semi-structured guide. Unclear how many participants pr. focus group, but 25 healthcare professionals who had completed one or more trainings, were included in the focus groups.	Thematic analysis	Adoption and integration of mindfulness practices in the workplace are feasible, but vary significantly by type, situation, and the individual. Perceived value, perceived skilfulness and integration within pre-existing life and work routine affected motivation and mindfulness practice at work. Participants adopted four basic models of adopting mindfulness -planned practice, episodic practice, on-the fly practice, contagion practice. Healthcare workers relied more on informal than on formal meditation practice (p. 240).
Irving et al. (2014) Canada	To explore if and how participation in a modified version of MBSR was perceived to be beneficial for healthcare professionals and the processes through which this may have occurred. Challenges to engagement in the program were also examined.	**Population:** 27 healthcare professionals (27 % physicians, 15 % psychologist, 15 % nurses, 8 % social workers, 8 % counsellors and 27 % complementary healthcare providers) **Speciality:** not specified. **Experience:** no info. Sex**:** 81 % female. **Age:** mean age 51 (23–82).	**MBI**: Adopted version of MBSR, 8 sessions, 2.5 h per week, 1-day silent retreat. The program included exercises emphasis on interpersonal mindfulness and communication skills. **Home practice:** mindfulness between sessions, not specified how much. **Location**: no info. **Instructor:** a psychologist and a physician, trained in teaching mindfulness at Massachusetts Center for Mindfulness.	Qualitative design (grounded theory). Six focus group interviews with 3–6 healthcare professionals. The interviews were conducted 3 weeks after completing the MBI.	Grounded theory analysis using axial and selective coding	Highlighted themes included becoming aware of perfectionism, the automaticity of "other focus" and the "helping or fixing mode". The findings illustrate the change process undertaken by participants and the implications across professionals and personals domains (p. 60).
Foureur et al. (2013) Australia	To pilot the effectiveness of an adopted MBSR intervention on the psychological wellbeing of nurses and midwives. Participants were also invited to take part interviews or focus groups to discuss their experiences of the program and their ongoing mindfulness practice.	**Population:** 20 nurses and 20 midwives **Speciality:** not specified. **Experience:** no info. Sex**:** 100 % female. **Age:** no info.	**MBI:** Adapted MBSR, one-day workshop. **Home practice:** 20 min per day for 8 weeks. **Location**: no info. **Instructor**: one of the researchers, who were committed to practice mindfulness (experienced psychologist)	Mixed method. Qualitative data consist of 2 individual interviews and 1 focus group interview with 8 of the participants.	Content analysis	The qualitative findings support this acceptability of this intervention. The content analysis revealed a range of enablers and barriers to incorporating mindfulness practice into the business of life (p. 114).
Cohen-Katz et al. (2005) USA	The effects of MBSR on nurse stress and burnout were studied and presented in a 3-part series. Part 3 highlights the qualitative data.	**Population:** 25 healthcare professions (90% were nurses, the rest was persons employed in pastoral care, respiratory therapy, and social work) **Speciality:** not specified. **Experience:** average 22 years of practice experiences Sex**:** 100 % female. **Age:** mean age 46,5.	**MBI:** MBSR, 8 sessions, 2.5 h per week, plus 1-day 6-hour silent retreat. **Home practice**: 45–60 min. mindfulness practice daily between sessions. **Location:** no info. I**nstructor**: certified MBSR teacher.	Mixed methods. Qualitative data consist of documents evaluation forms, e-mails, 4 in-depth interviews with graduates from the MBSR program performed by a newsletter journalist, 2 interviews with the Vice President for Clinical Services (third author) performed by the first author, and one focus group with 7 participants from the MBSR program performed by a moderator.	Thematic analysis, based on a 32-item codebook, developed by the research team	Nurses found MBSR helpful. Greater relaxation, self-care and improvement in work- and family relationships were described as benefits. Challenges included restlessness, physical pain, and dealing with difficult emotions (p. 78).

## 3 Results

### 3.1 Characteristics of the primary studies

Eighteen studies were included in the meta-synthesis (Table 3). The primary aim in three of the studies was implementation of mindfulness in hospital settings (Byron et al., 2015; Lebares et al., 2020; Mealer et al., 2017). The remaining studies had a broader focus on healthcare professionals’ experiences with mindfulness training, but they also included information about facilitating and inhibiting factors to mindfulness implementation in the Results section. The interventions consisted of adapted versions of Mindfulness-Based Stress Reduction (*n* = 11), adapted versions of Mindfulness-Based Cognitive Therapy (*n* = 3), and other mindfulness-based interventions, which included formal and informal mindfulness practices (*n* = 4). On average, the interventions included 14.9 h (range: 1–26 h) of teacher-led training from 1 day to 8 weeks. Home practice in between the teacher-led training was included in 15 studies. All studies except one that also offered mindfulness training through videoconference were delivered as in-person group training (Muir and Keim-Malpass, 2020). Nine studies were conducted at a hospital, two at a university or campus, and seven did not specify the setting. Healthcare professionals (*n* = 410) with direct patient care provided qualitative data from focus groups or individual interviews. Eleven studies used a qualitative design with individual interviews (*n* = 7) or focus groups (*n* = 4), and seven studies used a mixed methods design, including individual interviews (*n* = 4) or both focus groups and individual interviews (*n* = 3). Some studies included healthcare professionals from a single profession; i.e., physicians (*n* = 4), nurses (*n* = 4), and midwives (*n* = 1), and the rest included healthcare professionals from different professions (*n* = 10). The healthcare professionals worked within a wide range of medical disciplines. The majority of participants were women ranging from 50 % to > 90 % in most studies (*n* = 13). Some studies (*n* = 5) did not provide data on sex. The clinical experience of the healthcare professionals ranged from newly-graduated to senior practitioner.

### 3.2 Essential principles of successful mindfulness implementation

Based on the included studies, we identified three main themes and 10 subthemes essential to successful implementation of mindfulness in hospital settings: 1) *Buying In,* 2) *Allocating time and space*, and 3) *Keeping it going*. The subthemes describe facilitating and inhibiting factors of importance for the implementation process, as illustrated in Table 4 and Supplementary Material File 6. These factors are often contiguous in the sense that the facilitating factors offer solutions to the inhibiting factors.

**Table 4:** Inhibiting and facilitating factors for implementing mindfulness in hospital setting. *Supplementary Material File 6 contain a Table with all references.

Review findings	Number of contributing studies*		Number of contributing studies*
**Theme 1: Buying in**			
*Inhibiting factors*		*Facilitating factors*	
Prejudices and misconceptions about mindfulness	3	Emphasize the value of mindfulness training to the work specific practice, including past participants experience and the evidence base of the effectiveness of mindfulness-based interventions	4
Down-prioritizing or feeling guilty of self-care	6	Associate the training with better patient care	2
Concerns about showing vulnerability in front of peers	2	Attending to inter-personal relationships in the local context when composing the groups	3
Physical or emotional challenges with practicing mindfulness	7	Creating a safe space, preparing for potential discomfort, making participation optional, and leaving room for different levels of engagement	4
**Theme 2: Allocating time and space**			
*Inhibiting factors*		*Facilitating factors*	
Lack of time	9	Leadership support in terms of securing protected time during working hours to attend mindfulness training and secure personal coverage	3
Space for engaging in mindfulness practice	3	Offering mindfulness training in-house but with the possibility to participate online if physical attendance is an obstacle for participating	2
**Theme 3: Keeping it going**			
*Inhibiting factors*		*Facilitating factors*	
Gaining knowledge from a book	1	Getting firsthand experiences with mindfulness including learning informal mindfulness practices, which can be integrated directly in clinical practice	14
Changing habits and sustaining the benefits of mindfulness training	7	Follow-up training	9
		Offering mindfulness training in a group. The group training is supportive, helps with overcoming challenges and applying it to clinical practice	10
		Identifying committed local champions serving to diffuse mindfulness through the implementation process	1

#### 3.2.1 Buying in

*Buying In* was identified as the first step of the implementation process. The results from the included studies described cultural values and held beliefs, which might influence buy-in to attend mindfulness-based interventions and engage in mindfulness practice (Aeschbach et al., 2021; Lebares et al., 2020; Lyddy et al., 2016; Weisbaum, 2021). Four subthemes were identified: *Emphasizing the value of mindfulness training, Associating self-care with better patient care, Attending to inter-professional relationships, and Creating a safe space.*

##### 3.2.1.1 Emphasizing the value of mindfulness training

Some healthcare professionals described concerns about prejudices, such as mindfulness being esoteric, not fitting into a rational worldview, mindfulness being only for those who had failed in their jobs, and worries about being ridiculed by peers for participating in mindfulness training (Aeschbach et al., 2021). Lebares et al. (2020) also described a conflict between wellbeing interventions and surgical identities and limited awareness of the evidence supporting mindfulness effectiveness (Lebares et al., 2020).

“*One participant reported that he was initially worried that mindfulness was something esoteric that would not fit into his rational worldview. Another resident physician stated that he subscribed to a stereotype of mindfulness as only being something for those who had failed in their job*” (Page 5) (Aeschbach et al., 2021).

Several studies offered solutions to address obstacles and facilitate buy-in. This included emphasizing past participants' experiences of the perceived value of mindfulness training, relating the use of mindfulness to the work-specific practice, and introducing evidence documenting the effectiveness of mindfulness-based interventions (Byron et al., 2015; Lebares et al., 2020; Mealer et al., 2017; Weisbaum, 2021):

“*We addressed cultural barriers by focusing on quantitative outcome measures that were meaningful to stakeholders, by crafting grand rounds presentations and elevator talks that specifically targeted the MBI* (Mindfulness-based Intervention) *evidence-base and by early beta-testing of ESRT* (Enhanced Stress Resilience Training) *among thought- and opinion-leaders in our department. This process transformed value, evidence, and experience into enabling influences*” (page 331) (Lebares et al., 2020).

##### 3.2.1.2 Associating self-care with better patient care

Several studies described that healthcare professionals tended to feel guilty about taking time out and prioritizing self-care (Cohen-Katz et al., 2005; Hunter et al., 2018; Irving et al., 2014). However, Lebares described that because of the commitment to put patients' needs first, a powerful facilitating factor to enhance buy-in was to associate self-care with better patient care when introducing mindfulness to surgeons (Lebares et al., 2020). In line with this, Weisbaum expressed that some physicians were interested in gaining knowledge, so they could introduce mindfulness to their patients, and, therefore, the study recommended including this information in the introductory program (Weisbaum, 2021):

“*And I also thought overall like, if I am going to speak to my patients in the future about Mindfulness-Based practice, I thought it would be helpful for me to kind of get a better understanding of what it's about, and see some of the benefits of it*” (page 209) (Weisbaum, 2021).

As such, to enhance buy-in it would be relevant to not only present the value of the training to healthcare professionals, but also to highlight the impact it might have on patient care.

##### 3.2.1.3 Attending to inter-professional relationships

Worry about showing vulnerability, especially when training with colleagues from other professions and seniorities, was also described as an inhibiting factor for attending mindfulness training (Nissim et al., 2019; Slatyer et al., 2018).

“*The participants were concerned that allowing themselves to ‘open up’ and experience vulnerability in the group would diminish their ability to function effectively when they returned to work. A more pressing concern, however, was that ‘displaying vulnerability’ in the group would be perceived negatively by fellow group members because it would be inconsistent with the expectations of their team and of the organization at large to ‘keep a stiff upper lip*” (page 36) (Nissim et al., 2019).

While some studies indicated that group composition with healthcare workers from the same profession and with similar seniority was important (Mealer et al., 2017; Nissim et al., 2019; Slatyer et al., 2018; Weisbaum, 2021), other studies found that training with co-workers from other professions had a positive impact on inter-professional relationships and communication (Irving et al., 2014; Knudsen et al., 2021; Muir and Keim-Malpass, 2020). As such, what could be experienced as an inhibiting factor in some hospital settings might be a facilitating factor in others, and, therefore, it is important to be attentive of the inter-professional relationships in the local context when planning and composing the groups for mindfulness training.

##### 3.2.1.4 Creating a safe space

Some healthcare professionals experienced physical or emotional challenges when practicing mindfulness, such as restlessness, discomfort, and dealing with difficult emotions (Cohen-Katz et al., 2005; Irving et al., 2014; Negus and Grobler, 2021; Weisbaum, 2021). Experiences of discomfort and struggles might influence the success of mindfulness practice, but these challenges were most frequent during the first weeks of the program and tended to decline during the course of the program (Cohen-Katz et al., 2005; Weisbaum, 2021):

“*Participants describing an initial discomfort or challenge that evolves into either a comfort or deep engagement with the activity later on in the program*” (page 228) (Weisbaum, 2021).

Some studies offered concrete solutions for how to meet the emotional challenges that healthcare professionals can experience when starting mindfulness training. These involved the teacher providing a safe space with respect and confidentiality, preparing healthcare professionals for potential discomfort, and offering the possibility of opting out of exercises if needed (Irving et al., 2014; Nissim et al., 2019).

“*Additionally, our analyses yielded the finding that many participants experienced feelings of guilt when making efforts to engage in basic self-care. For this reason, MBSR* (Mindfulness-Based Stress Reduction) *instructors may wish to be particularly explicit about the potential for distress while learning mindfulness. They may choose to reassure participants about safeguards to confidentiality should they require additional support, and openly model and discuss appropriate help-seeking behaviors as an integral part of self-care*” (page 69) (Irving et al., 2014).

Furthermore, some studies found that making participation in the mindfulness training optional and leaving room to engage to whatever level they were comfortable facilitated buy-in to attend the training (Byron et al., 2015; Weisbaum, 2021).

*“Staff described the fact that participation was optional and offered as an invitation rather than an expectation facilitated their participation” (page 9)* (Byron et al., 2015).

#### 3.2.2 Allocating time and space

Allocating time and space for attending mindfulness sessions and practicing mindfulness was described as essential for a successful implementation process. This theme was divided into two subthemes: *Securing protected time* and *Providing in-house or online training*.

##### 3.2.2.1 Securing protected time

Time constraints were highlighted as an inhibiting factor in many of the studies (Byron et al., 2015; Irving et al., 2014; Knudsen et al., 2021; Lyddy et al., 2016).

“*I recognize the importance, but it is always about the time”; "there was no way I could ever commit”; "it's lack of time”; and "I was unable to attend because of the demand*” (page 9) (Byron et al., 2015).

Several studies emphasized that leadership support was important for healthcare professionals to engage in the training. This included securing protected time during work hours to attend and practice mindfulness (Byron et al., 2015; Lebares et al., 2020; Nissim et al., 2019) and prioritizing sufficient staff coverage, so the healthcare professionals were able to attend the sessions.

*The fact that the CPR-T* (Compassion, Presence, and Resilience Training) *was provided in-house and additional coverage was arranged so that participants could attend sessions during work hours was perceived by the participants not only as making it more feasible and convenient for them to attend but also as representing a much-needed organizational acknowledgment of job- related stress* (page 35) (Nissim et al., 2019)*.*

Lebares et al. (2021) found that although protected time to attend the mindfulness sessions was provided, some residents experienced tasks piling up after the class, which was a challenge (Lebares et al., 2020):

“*We discovered that the allocated "protected time" for this class […] involved held pages but not alternative service coverage. This resulted in senior residents encountering numerous ‘piled up’ tasks awaiting them after class […]. Additionally, they encountered the ire of impatient nurses and attendings. This resulted in a dissonant experience for them, being required to attend a class and being resented for doing so*” (page 332) (Lebares et al., 2020)

Thus, protected time must include true service coverage, but also buy-in from colleagues.

##### 3.2.2.2 Providing in-house or online training

In most of the studies, the mindfulness training was held at the hospital, making it easier for the healthcare professionals to attend. However, lack of space to practice, noise from the surroundings, and the shift from high speed to the mindfulness class could challenge practicing mindfulness at the departments (Byron et al., 2015). As such, in-house training might be preferable, but a physical space suitable for practice is necessary. In one study, the mindfulness training was offered both in-person and online, making it more flexible for healthcare professionals working at all hours of the day (Muir and Keim-Malpass, 2020):

“*The majority of participants participated in the sessions through a mix of formats, which suggests that offering online attendance in conjunction with in-person attendance may enhance participant motivation and adherence to the program*” (page 215) (Muir and Keim-Malpass, 2020).

Mealer et al. (2017) also recommended a hybrid delivery format, including online didactic content, teleconference components, and podcasts: "*The more online the better, it would increase participation*" (page 6) (Mealer et al., 2017).

#### 3.2.3 Keeping it going

Keeping it going involved factors related to sustaining mindfulness practice. The theme encompassed four subthemes: *Learning informal mindfulness practice, Offering group training, Offering follow-up, and Identifying local champions*. Many healthcare professionals expressed concerns about sustaining mindfulness after the intervention ended (Aeschbach et al., 2021; Cohen-Katz et al., 2005; Knudsen et al., 2021; Nissim et al., 2019). Nurses, who had already been practicing meditation, reported that the mindfulness training helped them increase their motivation to practice, but those who were new to meditation tended to discontinue the practice once their feeling of stress diminished (Nissim et al., 2019). However, several facilitating factors to support sustaining mindfulness practices were identified.

##### 3.2.3.1 Learning informal mindfulness practices

Most of the studies described that learning short informal mindfulness techniques, such as mindful walking, mindful eating, pausing, and breathing exercises, were important. It was easier to integrate these short practices into healthcare professionals’ daily routines and make them a habit, whereas the formal practice was challenging (Irving et al., 2014; Lyddy et al., 2016; Minichiello et al., 2020; Weisbaum, 2021).

“*They explained that it was much easier to engage in the ‘practical’ and ‘flexible and feasible’ micropractices than the ‘rigid’ sitting meditation. Likewise, whereas the formal sitting meditation practice was perceived as ‘something extra I had to go and do’, the informal practices (*e.g.*, ‘mindful eating’) merely involved doing ‘something I naturally do every day’ in a more mindful way […] the micropractices, which were utilized ‘on demand,’ were associated with an immediate relief of stress, thus motivating the participants to ‘stick to’ them, ‘internalize,’ and ‘make them into a habit*” (page 39) (Nissim et al., 2019).

Healthcare professionals described that these informal practices had an immediate effect, such as feeling grounded, less stressed, more focused, calmer, and more present and compassionate towards patients and colleagues (Aeschbach et al., 2021; Hunter et al., 2018; Knudsen et al., 2021; Nissim et al., 2019). Several studies pointed out that informal practice was not enough (Aeschbach et al., 2021; Brun et al., 2023; Irving et al., 2014; Lyddy et al., 2016) and "*formal practice made it easier to engage in informal practice*" (page 65) (Irving et al., 2014). In one study, the intervention group received 26 h of mindfulness training, while the control group received only written material. The researchers found that gaining knowledge about mindfulness from a book had minor or no effect and that healthcare professionals were not able to implement this knowledge in their daily lives. Thus, first-person experiences were necessary to build mindfulness skills (Aeschbach et al., 2021).

##### 3.2.3.2 Offering group training

In many of the studies, the authors described the group training as supportive, making participants feel more connected and less alone with their challenges (Byron et al., 2015; Irving et al., 2014; Knudsen et al., 2021; Lyddy et al., 2016).

“*The group also provided a safe space for discussion, and motivated participants to practice and keep going when they came across challenges; And if there was someone quite closed to it, or if I was closed to it, then there's always someone there to challenge you… So I think it's being in the group and doing the course, helps you to continue*” (page 1234) (Hunter et al., 2018).

The group training helped healthcare professionals overcome internal challenges, normalizing difficult emotions, motivated them to practice mindfulness and carry it into clinical practice, recognizing that positive relationships with co-workers were fundamental for improving the work culture (Byron et al., 2015; Hunter et al., 2018; Irving et al., 2014).

“*Participants described what we will call a mutual mindful experience which encouraged staff receptiveness to this new approach through the common experience of going through the training sessions together and engaging in daily practice. This direct collective and individual experience created a culture of mindfulness to facilitate the implementation process and long-term sustainability of the initiative*” (page 6) (Byron et al., 2015).

##### 3.2.3.3 Offering follow-up

Forming supportive networks (Byron et al., 2015; Cohen-Katz et al., 2005; Lebares et al., 2020; Muir and Keim-Malpass, 2020) and providing follow-up training or monthly drop-in groups to facilitate sustaining a mindfulness practice was suggested by many of the healthcare professionals (Brun et al., 2023; Foureur et al., 2013; Irving et al., 2014; Nissim et al., 2019).

“*Several informal networks were created in the hospital system as an outlet for nurses to maintain their practice. Prior to this project, a nurse graduate from our ongoing treatment group formed a monthly peer support group, in which meditation tapes are played and discussions are held about the practice. […] Twice a week, they practice a 20-minute meditation during their lunch hour*” (page 85) (Cohen-Katz et al., 2005).

##### 3.2.3.4 Identifying local champions

Researchers in one study, which explicitly explored the implementation process, described the importance of having committed local champions acting as early adopters serving to diffuse mindfulness through communication and influence. These champions enhanced not only buy-in of the staff but also long-term adherence (Byron et al., 2015).

“*One of the clinical leaders who had previous knowledge and personal experience with mindfulness functioned in the role of a local champion, taking personal initiative, and taking an active role in planning and implementing the training sessions. Her dedication to the project motivated others to become involved*” (page 13) (Byron et al., 2015).

The implementation process was described as staff gradually becoming more inquisitive and receptive to the concept, which had a contagion effect, where the observation of mindfulness in practice enabled others to buy-in to it and accept mindfulness as a part of the organizational culture (Byron et al., 2015).

## 4 Discussion

In this meta-synthesis, we identified several facilitating and inhibiting factors of importance for successful implementation of mindfulness in hospital settings. In the following, we discuss the three overall themes – *Buying In, Allocating time and space,* and *Keeping it going* with reference to the i-PARIHS framework and existing literature. I-PARIHS is a multidimensional conceptual framework that can help explain and understand the complexity involved in the uptake of evidence into practice. The framework provides important theoretical perspectives to guide implementation processes, and therefore it can add further explanations to our results (Harvey and Kitson, 2015; Harvey and Kitson, 2016).

### 4.1 Buying in

The i-PARIHS framework describes several important elements to consider in implementation processes. The first element involved in a successful implementation is awareness of the evidence about the innovation being implemented, including the relative advantages compared to existing practice (Harvey and Kitson, 2016). In line with other studies (DeMauro et al., 2019; Hunter, 2016; Morgan et al., 2015; Wu et al., 2021), we found in this meta-synthesis that healthcare professionals experienced several advantages of the mindfulness training, such as feeling grounded, less stressed, more focused, calmer, and more present and compassionate towards patients and colleagues. Presenting the evidence and experiences from past participants was the first important step in the implementation process. Rigorous evidence, however, is rarely enough to guarantee uptake into practice. This process is subject to negotiation, contestation, and adaptation before it becomes implemented (Harvey and Kitson, 2015; Harvey and Kitson, 2016). Therefore, according to i-PARISH, a second factor to consider is related to the recipients, who are influenced by the implementation at both the individual and collective levels (Harvey and Kitson, 2015). An important question to explore is, whether healthcare professionals **want** to implement the innovation, in terms of fit with their existing practices, values and beliefs, and the view of their peers (Harvey and Kitson, 2015). We found that prejudices about mindfulness, feeling guilty about prioritizing self-care, and concerns about showing vulnerability in front of peers can be an inhibiting factor to buy-in to the concept. These points are supported by other studies (Micklitz et al., 2021; Morgan et al., 2015). Micklitz et al. (2021) found that promoting mindfulness training as professional development and enhance feeling permitted to practice self-care facilitated investment and engagement with mindfulness practice, whereas lack of explicit support from supervisors might discourage employees from attending mindfulness-based interventions (Micklitz et al., 2021). In addition, they found that in organizations where participants were concerned about being seen as weak and vulnerable, they might not share their struggles, but in organizations where they feel safe to share difficult emotions with others, practicing mindfulness with peers had the potential to enhance greater acceptance and compassion (Micklitz et al., 2021). These perspectives point to the importance of creating a safe space and being attentive to inter-professional relationships in the local context when planning and composing the groups for mindfulness training. In context with high psychological safety, training mindfulness with interdisciplinary colleagues might be a facilitator for implementing mindfulness in the department. However, if this is not the case, training with disciplinary colleagues is preferred.

### 4.2 Allocating time and space

Allocating time and space for attending mindfulness sessions and practicing mindfulness was described as essential for the implementation process. According to the i-PARIHS framework, successful implementation depends on several context-related factors, such as leadership support and organization priorities (Harvey and Kitson, 2016). This resonates with our results showing that leadership support is important in terms of securing protected time during working hours to attend and to practice mindfulness, staff coverage, and allocating a room close to the workplace. These perspectives are supported by studies describing implementation of mindfulness, albeit in other work settings. In a case study exploring implementation of mindfulness in secondary schools, researchers found that having support from management and allocation of sufficient time and financial resources was essential to enable implementation. – “*if you're going to do this you've got to resource it properly and understand there is a commitment required there”* p. 383 (Wilde et al., 2019). Micklitz et al. (2021) pointed out that, in a context where employees are under a lot of pressure, adding mindfulness training might exacerbate feelings of stress, lead to inability to practice mindfulness and dropping out (Micklitz et al., 2021). However, protected time and being released from their work to attend training sessions made employees feel permitted to practice self-care, which facilitated investment in the mindfulness training (Micklitz et al., 2021).

### 4.3 Keeping it going

We found that sustaining mindfulness after the intervention ended was a challenge. However, several facilitating factors to support sustaining the mindfulness practices were identified. This included getting firsthand experiences from formal mindfulness practice, combined with learning informal practices, which could be more easily integrated into clinical practice. However, the mindfulness-based interventions in this study varied greatly, and the most effective duration and delivery form to enhance lasting benefits were unclear. Previous meta-analyses’ have shown inconsistent results regarding the optimal form and delivery of mindfulness-based interventions (Lomas et al., 2019; Spinelli et al., 2019; Vonderlin et al., 2020). Vonderlin et al. (2020) found that neither the type of mindfulness-based intervention nor aspects of delivery significantly affected the outcome (Vonderlin et al., 2020), but Lomas et al. (2019) reported larger effects on health-related outcomes for standardized versions of Mindfulness-Based Stress Reduction (Lomas et al., 2019). In addition, Vonderlin et al. (2019) found that although duration of the program in weeks did not directly relate to the effectiveness, hours of attendance were significantly associated with higher improvements in mindfulness, wellbeing, and reduction of burnout symptoms (Vonderlin et al., 2020). This supports the assumption that sustaining mindfulness requires a certain amount of practice. More knowledge to determine optimal duration and amount of practice is therefore needed.

Other context-related factors related to successful implementation according to the i-PARIHS framework encompass whether the work environment supports ongoing learning and opportunities to create supportive networks and feedback processes (Harvey and Kitson, 2015; Harvey and Kitson, 2016). This resonates with the results that group training with colleagues helped overcome challenges with training mindfulness and made it easier to carry it out to clinical practice and engage in daily practice. Forming supportive networks and offering follow-up sessions were also valuable in building a community of mindfulness practice and enhance sustainability.

As discussed above, implementation of mindfulness in hospitals is a complex process, and according to the i-PARIHS framework, the core construct of this process is facilitation. Successful implementation depends upon the ability of a facilitator and the facilitation process to enable people to adopt the innovation within their context. Facilitation can involve one or more individuals, who apply a combination of strategies to enable and support change (Harvey and Kitson, 2015; Harvey and Kitson, 2016). In line with this, we found that it was important to have committed local champions, who acted as early adopters and served to diffuse mindfulness through communication and influence. This point is echoed in a study about implementation of Mindfulness-Based Cognitive Therapy to people in risk of recurrent depression (Rycroft-Malone et al., 2019). The authors interviewed stakeholders (clinicians in management, mindfulness-teachers, and service users) and found that the most important facilitator was the role of a skillful and resourceful mindfulness practitioner, who championed bottom-up implementation, with top-down support and investments (Rycroft-Malone et al., 2019). The most successful implementations had more than one champion, often at different levels in the organization. Therefore, local champions who have the skills and resources to facilitate activities to support mindfulness in hospital settings might be decisive for successful implementation. However, this was described in only one of the included studies, and more research is required.

### 4.4 Limitations and strengths

There are limitations to consider when interpreting the results of this meta-synthesis. First, 16 of the included studies were from Western countries and nine of these were from the United States of America and Canada, which might affect the transferability to countries with organizational healthcare structures that are not comparable.

Second, although all the studies described qualitative data on factors affecting implementation of mindfulness in hospital settings, their contribution to this meta-synthesis varied, and only three studies had implementation as their primary focus. The rest focused on healthcare professionals’ experiences with mindfulness training more generally.

Third, there is a risk of selection bias, since all included studies used self-selected samples. Therefore, the included healthcare professionals represent a motivated group, which might affect the result positively. We might have been able to identify more inhibiting factors if healthcare professionals who declined participation in mindfulness training had been interviewed. In addition, in 12 of the studies, most of the participants were female, and five of the studies did not describe data on sex.

Fourth, we narrowed the inclusion criteria to mindfulness-based interventions with a focus on training formal and informal mindfulness practices with a qualified mindfulness teacher, but the intervention structure, length, and delivery varied. This means that we could not determine which type of intervention was best. More knowledge of mindfulness-based interventions developed specifically to fit healthcare professionals working in hospital settings is needed.

Fifth, the interviews in most of the studies were conducted shortly after the course ended with no follow-up data. Since other studies have reported that participants tend to reduce mindfulness training over time (Kriakous et al., 2021; Spinelli et al., 2019), more knowledge about sustainability and long-term implementation of mindfulness is needed.

Finally, most of the studies had limitations regarding description of the relationship between researcher and participants and ethical issues (Critical Appraisal Skills Programme, 2018). Often the studies reported a minimum of ethical considerations, such as informed consent and formal ethical approval, but nothing further. Mindfulness-based interventions are psychological interventions, which involve teaching participants to observe mental, emotional, and bodily responses to everyday life situations. Ethical considerations of how to ensure a safe learning space and how participants should respond if they experience adverse effects of the training would be relevant to describe in future studies.

There are also several strengths to the study. As a multidisciplinary team of reviewers, we conducted this qualitative meta-synthesis following a protocol and a rigorous method with comprehensive literature searches in six databases. Multiple researchers were engaged in screening, quality assessment, data extraction, and interpreting the results. We included data from healthcare professionals from different professions, working within a wide range of medical disciplines. Thus, this meta-synthesis described a range of experiences concerning factors affecting mindfulness implementation within different hospital settings.

## 5 Conclusion

From this meta-synthesis, we have contributed knowledge of factors affecting successful implementation of mindfulness in hospital settings. Implementing mindfulness in hospital settings is a complex process involving various factors related to cultural values, held beliefs about mindfulness, inter-professional relationships at the workplace, and context-related factors, such as time and space. Successful implementation might depend upon the facilitating process of enabling healthcare professionals to adopt the innovation within their context. Therefore, local champions who have the skills and resources to facilitate activities to support mindfulness in hospital settings might be decisive for successful implementation. However, few of the included studies described specific information about the facilitation process. More research on facilitation, sustainability, and long-term implementation is required.

## Recommendations for clinical practice

Based on the identified results about facilitating factors for mindfulness implementation, we formulated eight recommendations for clinical practice (Table 5), which can help guide management and stakeholders in the planning process to enhance the likelihood of a successful implementation of mindfulness in hospital settings.

**Table 5:** Recommendations for Clinical Practice.

Theme	Recommendations for Clinical Practice
Buying In	1. Present the evidence of mindfulness-based interventions, the perceived value and relevance to the work specific context, and associate mindfulness training with better patient care. 2. Make participation optional. 3. Offer mindfulness training in groups and be sensitive to the inter-personal relationships in the local context when composing the group.
Allocating time and space	4. Secure protected time and personal coverage and allocate a room close to the workplace to make it possible for healthcare professionals to attend mindfulness training. 5. Provide the possibility to participate online if physical attendance is an obstacle for participating.
Keeping it going	6. Offer guidance in short informal mindfulness practices, which can be integrated directly into the work setting. 7. Form supportive networks and follow-up training. 8. Identify committed local champions serving to diffuse knowledge of mindfulness trough the implementation process.

## Ethical approval and informed consent

This study is based on qualitative data from other studies and therefore ethical approval and informed consent are not applicable.

## Funding

This work was financially supported by a scholarship from the Graduate School, Faculty of Health Sciences, University of Southern Denmark, the Department of Gynecology and Obstetrics, Horsens Regional Hospital and the Department of Cardiology, Lillebaelt Hospital, Southern Denmark. External funding was provided by the Health Research Foundation of Central Denmark Region (A3629).

Randi Karkov Knudsen: None conflicts of interest

Sine Skovbjerg: None conflicts of interest

Elna Leth Pedersen: None conflicts of interest

Camilla Littau Nielsen: None conflicts of interest

Marie Højriis Storkholm: None conflicts of interest

Connie Timmermann: None conflicts of interest

## CRediT authorship contribution statement

**Randi Karkov Knudsen:** Writing – original draft, Visualization, Validation, Project administration, Methodology, Investigation, Funding acquisition, Formal analysis, Data curation, Conceptualization. **Sine Skovbjerg:** Writing – review & editing, Validation, Supervision, Methodology, Formal analysis, Conceptualization. **Elna Leth Pedersen:** Writing – review & editing, Validation, Methodology, Investigation, Formal analysis, Conceptualization. **Camilla Littau Nielsen:** Writing – review & editing, Validation, Methodology, Investigation, Formal analysis, Conceptualization. **Marie Højriis Storkholm:** Writing – review & editing, Methodology, Funding acquisition, Conceptualization. **Connie Timmermann:** Writing – review & editing, Validation, Supervision, Methodology, Investigation, Formal analysis, Conceptualization.

## Declaration of competing interest

The authors declare that they have no known competing financial interests or personal relationships that could have appeared to influence the work reported in this paper.

## Appendix Supplementary materials

Image, application 1
