# Supplementary material for: Factors affecting implementation of mindfulness in hospital settings: A qualitative meta-synthesis of healthcare professionals' experiences
Source: Int J Nurs Stud Adv. 2024 Mar 27;6:100192. doi: 10.1016/j.ijnsa.2024.100192 (PMC11080443; doi:10.1016/j.ijnsa.2024.100192)
Supplement: Supplementary file 1 [file mmc1.docx]

**Supplementary Material File 1**

ENTREQ: Enhancing Transparency in Reporting the Synthesis of Qualitative Research

| **Number and item.** | **Guide and description** | **Reported on page** |
| --- | --- | --- |
| 1. Aim | State the research question the synthesis addresses. | 5 |
| 2. Synthesis methodology | Identify the synthesis methodology or theoretical framework which underpins the synthesis, and describe the rationale for choice of methodology *(e.g. meta-ethnography, thematic synthesis, critical interpretive synthesis, grounded theory synthesis, realist synthesis, meta-aggregation, meta-study, framework synthesis).* | 5-6 |
| 3. Approach to searching | Indicate whether the search was pre-planned (*comprehensive search strategies to seek all available studies)* or iterative (*to seek all available concepts until they theoretical saturation is achieved)*. | 6 |
| 4. Inclusion criteria | Specify the inclusion/exclusion criteria *(e.g. in terms of population, language, year limits, type of publication, study type).* | 7 |
| 5. Data sources | Describe the information sources used (e.g. *electronic databases (MEDLINE, EMBASE, CINAHL, psycINFO, Econlit), grey literature databases (digital thesis, policy reports), relevant organisational websites, experts, information specialists, generic web searches (Google Scholar) hand searching, reference lists)* and when the searches conducted; provide the rationale for using the data sources. | 6 |
| 6. Electronic Search strategy | Describe the literature search *(e.g. provide electronic search strategies with population terms, clinical or health topic terms, experiential or social phenomena related terms, filters for qualitative research, and search limits)*. | 6-7 Supplementary file 2 |
| 7. Study screening methods | Describe the process of study screening and sifting *(e.g. title, abstract and full text review, number of independent reviewers who screened studies).* | 8 |
| 8  Study characteristics | Present the characteristics of the included studies *(e.g. year of publication, country, population, number of participants, data collection, methodology, analysis, research questions).* | 15-19 |
| 9. Study selection results | Identify the number of studies screened and provide reasons for study exclusion *(e,g, for comprehensive searching, provide numbers of studies screened and reasons for exclusion indicated in a figure/flowchart; for iterative searching describe reasons for study exclusion and inclusion based on modifications t the research question and/or contribution to theory development).* | 9 |
| 10. Rationale for appraisal | Describe the rationale and approach used to appraise the included studies or selected findings *(e.g. assessment of conduct (validity and robustness), assessment of reporting (transparency), assessment of content and utility of the findings).* | 10-11 |
| 11. Appraisal items | State the tools, frameworks and criteria used to appraise the studies or selected findings *(e.g. Existing tools: CASP, QARI, COREQ, Mays and Pope* [[25](https://bmcmedresmethodol.biomedcentral.com/articles/10.1186/1471-2288-12-181#ref-CR25)]*; reviewer developed tools; describe the domains assessed: research team, study design, data analysis and interpretations, reporting).* | 10-11 |
| 12. Appraisal process | Indicate whether the appraisal was conducted independently by more than one reviewer and if consensus was required. | 10 |
| 13. Appraisal results | Present results of the quality assessment and indicate which articles, if any, were weighted/excluded based on the assessment and give the rationale. | 11 |
| 14. Data extraction | Indicate which sections of the primary studies were analysed and how were the data extracted from the primary studies? *(e.g. all text under the headings “results /conclusions” were extracted electronically and entered into a computer software).* | 12 |
| 15. Software | State the computer software used, if any. | 12 |
| 16. Number of reviewers | Identify who was involved in coding and analysis. | 12 |
| 17. Coding | Describe the process for coding of data *(e.g. line by line coding to search for concepts).* | 12 |
| 18. Study comparison | Describe how were comparisons made within and across studies *(e.g. subsequent studies were coded into pre-existing concepts, and new concepts were created when deemed necessary).* | 12 |
| 19. Derivation of themes | Explain whether the process of deriving the themes or constructs was inductive or deductive. | 12, supplementary file 5 |
| 20. Quotations | Provide quotations from the primary studies to illustrate themes/constructs, and identify whether the quotations were participant quotations of the author’s interpretation. | 20-28, Supplementary file 5 |
| 21. Synthesis output | Present rich, compelling and useful results that go beyond a summary of the primary studies (e.g. *new interpretation, models of evidence, conceptual models, analytical framework, development of a new theory or construct).* | 20-28, 36 |

* Reference: Tong A, Flemming K, McInnes E, Oliver SA, Craig J. Enhancing transparency in reporting the synthesis of qualitative research: ENTREQ. BMC Medical Research Methodology 2012, 12:181.

| **Scopus 27 June, 2023** | |
| --- | --- |
| **PEO** | **Keywords** |
| Population (Healthcare professionals) | (TITLE-ABS-KEY ((("healthcare OR "health care") W/2 (worker* OR professional* OR employee* OR personnel"))) OR TITLE-ABS-KEY ("hospital staff" OR "health personnel" OR nurs* OR midwi* OR physician* OR clinician* OR doctor*) |
| **AND** |  |
| Exposure (MBIs) | TITLE-ABS-KEY (Mindful* OR meditation OR mbsr OR mbi) |
| **AND** |  |
| Outcome (Experience, qualitative research) | TITLE-ABS-KEY (Qualitative OR interview* OR "focus group" OR "focus groups" OR experience* OR ethno*) |
|  | Search results: 2726 |

**Supplementary Material File 2:** Search strategy in all databases

| **Scopus 27 June 2023** | |
| --- | --- |
| **PEO** | **Keywords** |
| Population (Healthcare professionals) | (TITLE-ABS-KEY ((("healthcare OR "health care") W/2 (worker* OR professional* OR employee* OR personnel"))) OR TITLE-ABS-KEY ("hospital staff" OR "health personnel" OR nurs* OR midwi* OR physician* OR clinician* OR doctor*) |
| **AND** |  |
| Exposure (MBIs) | TITLE-ABS-KEY (Mindful* OR meditation OR mbsr OR mbi) |
| **AND** |  |
| Outcome (Experience, qualitative research) | TITLE-ABS-KEY (Qualitative OR interview* OR "focus group" OR "focus groups" OR experience* OR ethno*) |

| **PsycInfo 27 June 2023** | |
| --- | --- |
| **PEO** | **Keywords** |
| Population (Healthcare professionals) | health personnel/ OR exp Nurses/ OR exp Physicians/ OR ((healthcare or health care) adj2 (worker* or professional* or employee* or personnel)).ab,id,ti. OR (hospital staff or health personnel or nurs* or midwi* or physician* or clinician* or doctor*).ab,id,ti. |
| **AND** |  |
| Exposure (MBIs) | exp Mindfulness/ OR exp Meditation/ OR (Mindful* or meditation or mbsr or mbi).ab.id.ti. |
| **AND** |  |
| Outcome (Experience, qualitative research) | exp Qualitative Methods/ OR exp interviews/ OR (Qualitative or interview* or "focus group" or "focus groups" or experience* or ethno*).ab,id,ti. OR (interview* or experience*).af. or qualitative.tw. |
|  | Search results: 2067 |

| **Pubmed 27 June 2023** | |
| --- | --- |
| **PEO** | **Keywords** |
| Population (Healthcare professionals) | (“Health Personnel”[MeSH Terms] OR “Nurses”[MESH Terms] OR “Nurse Midwives”[MeSH Term] OR “Physicians”[MESH Term] OR (“healthcare professional”[Title/Abstract:~2] OR “health care professional””[Title/Abstract:~2] OR “healthcare professionals”[Title/Abstract:~2] OR “health care professionals”[Title/Abstract:~2] OR “healthcare worker”[Title/Abstract:~2] OR “health care worker”[Title/Abstract:~2] OR “healthcare workers”[Title/Abstract:~2] OR “health care workers”[Title/Abstract:~2] OR “healthcare employee”[Title/Abstract:~2] OR “health care employee”[Title/Abstract:~2] OR “healthcare employees”[Title/Abstract:~2] OR “health care employees”[Title/Abstract:~2] OR “healthcare personnel”[Title/Abstract:~2] OR “health care personnel””[Title/Abstract:~2] OR (“Health Personnel”[Text Word] OR “hospital staff”[Text Word] OR “nurs*”[Text Word] OR “midwi*”[Text Word] OR “physician*”[Text Word] OR “clinician*”[Text Word] OR “doctor*”[Text Word])) |
| **AND** |  |
| Exposure (MBIs) | (“Mindfulness” [MeSH Term] OR “Meditation”[MeSH Term] OR “(“mindful*”[Text Word] OR “Meditation”[Text Word] OR “mbsr”[Text Word] OR “mbi”[Text Word])) |
| **AND** |  |
| Outcome (Experience, qualitative research) | (“Qualitative research”[MeSH Term] OR (“interview”[Publication Type] OR “intervies as Topic”[MeSH Term]) OR (“Qualitative”[Text Word] OR “interview*[Text Word] OR "focus group"[Text Word] OR "focus groups"[Text Word] OR experience*[Text Word] OR ethno*[Text Word]) OR ((“interview*”[All Fields] OR “experience*”[*All Fields] OR”Qualitative”[Text Word])) |
|  | Search results: 1781 |
|  |  |

| **CINAHL 27 June 2023** | |
| --- | --- |
| **PEO** | **Keywords** |
| Population (Healthcare professionals) | (MH "Health Personnel") OR (MH "Midwives+") OR (MH "Nurses+") OR (MH "Physicians+") OR ((healthcare or health care) N2 (worker* or professional* or employee* or personnel)) OR TI ("health personnel" or “Hospital staff” OR nurs* or midwi* or physician* or clinician* or doctor* ) OR AB ("health personnel" OR “Hospital staff" or nurs* or midwi* or physician* or clinician* or doctor* ) |
| **AND** |  |
| Exposure (MBIs) | (MH "Mindfulness") OR (MH "Meditation") OR TI (Mindful* or meditation or mbsr or mbi) OR AB (Mindful* or meditation or mbsr or mbi) |
| **AND** |  |
| Outcome (Experience, qualitative research) | (MH “qualitative studies”) (MH “questionnaires+”) (MH “interviews+”) (MH “attitude+”) (MH “research, nursing”) (MH “ethnonursing research”) (MH “ethnological research”) (MH “ethnographic research”) (MH “observational methods”) (MH “phenomenological research”) (MH “phenomenology”) (MH “focus groups”) (MH “discourse analysis”) (MH “content analysis”) (MH “life experiences”) (MH “narratives”) OR TI ( Qualitative or interview* or "focus group" or "focus groups" or experience* or ethno* ) OR AB ( Qualitative or interview* or "focus group" or "focus groups" or experience* or Ethno*) |
|  | Search results: 1227 |

| **Web of Science 27 June 2023** | |
| --- | --- |
| **PEO** | **Keywords** |
| Population (Healthcare professionals) | **"healthcare professional" or "health care professional" or "healthcare professionals" or "health care professionals" or "healthcare worker” or "health care worker” or "healthcare workers” or "health care workers” or "healthcare employee” or "health care employee” or "healthcare employees" or “health care employees” or "health personnel" or "healthcare personnel" or "health care personnel" or "hospital staff" or nurs* or midwi* or physician* or clinician* or doctor*** (Topic) |
| **AND** |  |
| Exposure (MBIs) | **Mindful* or meditation or mbsr or mbi**(Topic) |
| **AND** |  |
| Outcome (Experience, qualitative research) | **Qualitative or interview* or "focus group" or "focus groups" or experience* or ethno*** (Topic) |
|  | Search results: 1719 |

| **ProQuest Dissertations & Theses Global 27 June 2023** | |
| --- | --- |
| **PEO** | **Keywords** |
| Population (Healthcare professionals) | noft (**"healthcare professional" or "health care professional" or "healthcare professionals" or "health care professionals" or "healthcare worker” or "health care worker” or "healthcare workers” or "health care workers” or "healthcare employee” or "health care employee” or "healthcare employees" or “health care employees” or "health personnel" or "healthcare personnel" or "health care personnel" or "hospital staff" or nurs* or midwi* or physician* or clinician* or doctor***) |
| **AND** |  |
| Exposure (MBIs) | noft (“**Mindful* or meditation or mbsr or mbi)** |
| **AND** |  |
| Outcome (Experience, qualitative research) | noft (**Qualitative or interview* or "focus group" or "focus groups" or experience* or ethno*** ) |
|  | Search results: 815 |

**Supplementary Material File 3:** Articles full text screening and reasons for exclusion

**Researcher who assessed the articles: Randi Karkov Knudsen and Camila Littau Nielsen**

#1100 - Crandall 2022

Mindful Self-Compassion Training and Nephrology Nurses' Self-Reported Levels of Self-Compassion, Burnout, and Resilience: A Mixed Methods Study X

Crandall, Jacqueline; Harwood, Lori; Wilson, Barbara; Morano, Catherine

Nephrology Nursing Journal 2022;49(5):405-417

Pitman, New Jersey American Nephrology Nurses' Association 2022

DOI: [10.37526/1526-744x.2022.49.5.405](https://dx.doi.org/10.37526/1526-744x.2022.49.5.405)

**Reasons for exclusion:** No qualitative data on factors affecting implementation of mindfulness in hospital settings

#456 - dosSantos 2016

Positive Effects of a Stress Reduction Program Based on Mindfulness Meditation in Brazilian Nursing Professionals: Qualitative and Quantitative Evaluation X

dos Santos, Teresa Maria; Kozasa, Elisa Harumi; Carmagnani, Isabel Sampaio; Tanaka, Luiza Hiromi; Lacerda, Shirley Silva; Nogueira-Martins, Luiz Antonio

Explore: The Journal of Science & Healing 2016;12(2):90-99

New York, New York Elsevier B.V. 2016

DOI: [10.1016/j.explore.2015.12.005](https://dx.doi.org/10.1016/j.explore.2015.12.005)

**Reasons for exclusion:** No qualitative data on factors affecting implementation of mindfulness in hospital settings

#6077 - Orellana-Rios 2017

Mindfulness and compassion-oriented practices at work reduce distress and enhance self-care of palliative care teams: A mixed-method evaluation of an "on the job" program X

Orellana-Rios, C. L.; Radbruch, L.; Kern, M.; Regel, Y. U.; Anton, A.; Sinclair, S.; Schmidt, S.

BMC Palliative Care 2017;17(1):

2017

DOI: [10.1186/s12904-017-0219-7](https://dx.doi.org/10.1186/s12904-017-0219-7)

**Reasons for exclusion:** No qualitative data on factors affecting implementation of mindfulness in hospital settings

#4671 - Couillet 2022

Issues of mindfulness implementation for oncology caregivers X

Couillet, A.; Malatier, M.; Le Bris, M. P.; Mastroianni, B.; Chvetzoff, G.

J Complement Integr Med Sep 1 2022;19(3):791-797

2022 Sep 1

DOI: [10.1515/jcim-2021-0372](https://dx.doi.org/10.1515/jcim-2021-0372)

**Reasons for exclusion:** Data based on survey

#540 - Darby 2017

Grist to the Mill: A Qualitative Investigation of Mindfulness-Integrated Cognitive Behaviour Therapy for Experienced Health Professionals X

Darby, Mick; Beavan, Vanessa

Australian Psychologist 2017;52(6):491-502

Philadelphia, Pennsylvania Taylor & Francis Ltd 2017

DOI: [10.1111/ap.12215](https://dx.doi.org/10.1111/ap.12215)

**Reasons for exclusion:** No qualitative data on factors affecting implementation of mindfulness in hospital settings

#325 - Turner 2013

A qualitative study examining the experiences of healthcare staff 12 months after their completion of an 8-week Mindfulness Based Stress Reduction course X

Turner, Ross

2013;(D.Clin.Psych.):N.PAG p-N.PAG p

University of Glasgow (United Kingdom) 2013

**Reasons for exclusion:** Wrong setting

#662 - Lehto 2018

Hospice and Palliative Care Provider Experiences With Meditation Using Mobile Applications x

Lehto, Rebecca H.; Heeter, Carrie; Allbritton, Marcel; Wiseman, Michelle

Oncology Nursing Forum 2018;45(3):380-388

Pittsburgh, Pennsylvania Oncology Nursing Society 2018

DOI: [10.1188/18.ONF.380-388](https://dx.doi.org/10.1188/18.ONF.380-388)

**Reasons for exclusion:** Wrong intervention (discussed with a third researcher)

#12914 - Banerjee 2017

A qualitative study with healthcare staff exploring the facilitators and barriers to engaging in a self-help mindfulness-based intervention. x

Banerjee, Moitree; Cavanagh, Kate; Strauss, Clara

Mindfulness / 2017;8(6):1653-1664

Germany SpringerGermany 2017 /

DOI: [10.1007/s12671-017-0740-z](https://dx.doi.org/10.1007/s12671-017-0740-z) **·** Ref ID: 2017-23671-001

**Reasons for exclusion:** Wrong intervention (self-help MBI - discussed with a third researcher)

#3357 - York 2007

A qualitative study into the experience of individuals involved in a mindfulness group within an acute inpatient mental health unit

York, M.

J Psychiatr Ment Health Nurs Sep 2007;14(6):603-8

2007 Sep

DOI: [10.1111/j.1365-2850.2007.01148.x](https://dx.doi.org/10.1111/j.1365-2850.2007.01148.x)

**Reasons for exclusion:** Wrong population

**Researcher who assessed the articles: Randi Karkov Knudsen and Elna Leth Pedersen**

#1157 - Ooms 2022

Enhancing the well-being of front-line healthcare professionals in high pressure clinical environments: A mixed-methods evaluative research project X

Ooms, Ann; Heaton-Shrestha, Celayne; Connor, Sarah; McCawley, Siobhan; McShannon, Jennie; Music, Graham; Trainor, Kay

International Journal of Nursing Studies 2022;132():N.PAG-N.PAG

Philadelphia, Pennsylvania Elsevier B.V. 2022

DOI: [10.1016/j.ijnurstu.2022.104257](https://dx.doi.org/10.1016/j.ijnurstu.2022.104257)

**Reasons for exclusion:** Wrong intervention

#1162 - Rao 2022

Health professionals' perspectives of integrating meditation into cardiovascular care: A descriptive qualitative study X

Rao, Angela; DiGiacomo, Michelle; Phillips, Jane L.; Hickman, Louise D.

Health & Social Care in the Community 2022;30(6):e4450-e4460

Malden, Massachusetts Wiley-Blackwell 2022

DOI: [10.1111/hsc.13849](https://dx.doi.org/10.1111/hsc.13849)

**Reasons for exclusion:** Wrong population

#4022 - Lynch 2018

Mantra meditation programme for emergency department staff: a qualitative study X

Lynch, J.; Prihodova, L.; Dunne, P. J.; O'Leary, C.; Breen, R.; Carroll, Á; Walsh, C.; McMahon, G.; White, B.

BMJ Open Sep 24 2018;8(9):e020685

2018 Sep 24

DOI: [10.1136/bmjopen-2017-020685](https://dx.doi.org/10.1136/bmjopen-2017-020685)

**Reasons for exclusion:** Wrong intervention (discussed with a third researcher)

#4805 - Rosen 2022

How Does Mindfulness Affect Registered Nurses Practicing in Acute Care Settings? A Study of the Lived Experiences of Nurses After Participating in a Mindfulness Program X

Rosen, J.; Penque, S.

J Am Psychiatr Nurses Assoc Jul 10 2022;():10783903221108767

2022 Jul 10

DOI: [10.1177/10783903221108767](https://dx.doi.org/10.1177/10783903221108767)

**Reasons for exclusion:** No qualitative data on factors affecting implementation of mindfulness in hospital settings

#705 - Valley 2018

A Thematic Analysis of Health Care Workers’ Adoption of Mindfulness Practices X

Valley, Morgan; Stallones, Lorann

WORKPLACE HEALTH & SAFETY 2018;66(11):538-544

Thousand Oaks, California Sage Publications Inc. 2018

DOI: [10.1177/2165079918771991](https://dx.doi.org/10.1177/2165079918771991)

**Reasons for exclusion:** Data based on survey

#12857 - Valley 2017

Feasibility of a mindfulness-based stress reduction intervention on health care safety. X

Valley, Morgan Anne

Dissertation Abstracts International: Section B: The Sciences and Engineering / 2017;78(3-B(E)):No-Specified

US ProQuest Information & LearningUS 2017 /

Ref ID: 2017-05712-032

**Reasons for exclusion:** Data based on survey

#4837 - TryggLycke 2022

Emergency Department Nurses' Experiences of a Mindfulness Training Intervention: A Phenomenological Exploration X

Trygg Lycke, S.; Airosa, F.; Lundh, L.

J Holist Nurs May 16 2022;():8980101221100091

2022 May 16

DOI: [10.1177/08980101221100091](https://dx.doi.org/10.1177/08980101221100091)

**Reasons for exclusion:** Wrong intervention (discussed with a third researcher)

#8345 - Verweij 2018

Does Mindfulness Training Enhance the Professional Development of Residents? A Qualitative Study X

Verweij, H.; van Ravesteijn, H.; van Hooff, M. L. M.; Lagro-Janssen, A. L. M.; Speckens, A. E. M.

Academic Medicine SEP 2018;93(9):1335-1340

2018 SEP

DOI: [10.1097/ACM.0000000000002260](https://dx.doi.org/10.1097/ACM.0000000000002260)

**Reasons for exclusion:** No qualitative data on factors affecting implementation of mindfulness in hospital settings

#866 - Horton‐Deutsch 2020

Moving from practice to praxis: A qualitative descriptive study revealing the value of Project7 Mindfulness Pledge©

Horton‐Deutsch, Sara; Monroe, Chelsie; Varney, Robert; Loresto, Figaro; Eron, Kathryn; Kleiner, Catherine

Journal of Nursing Management (John Wiley & Sons, Inc.) 2020;28(3):728-734

London, <Blank> Hindawi Limited 2020

DOI: [10.1111/jonm.12990](https://dx.doi.org/10.1111/jonm.12990)

**Reasons for exclusion:** Wrong intervention

#990 - Hedderman 2021

Mindfulness moments for clinicians in the midst of a pandemic X

Hedderman, E.; O'Doherty, V.; O'Connor, S.

Irish Journal of Psychological Medicine 2021;38(2):154-157

Cambridge University Press 2021

DOI: [10.1017/ipm.2020.59](https://dx.doi.org/10.1017/ipm.2020.59)

**Reasons for exclusion:** No qualitative data on factors affecting implementation of mindfulness in hospital settings

#4736 - Kim 2022

Role of an Online Skill-Based Mindfulness Program for Healthcare Worker's Resiliency During the COVID-19 Pandemic: A Mixed-Method Study X

Kim, S.; Crawford, J.; Hunter, S.

Front Public Health 2022;10():907528

2022

DOI: [10.3389/fpubh.2022.907528](https://dx.doi.org/10.3389/fpubh.2022.907528)

**Reasons for exclusion:** No qualitative data on factors affecting implementation of mindfulness in hospital settings

#1158 - Osman 2022

Using PhotoVoice to understand mindfulness in health care practitioners X

Osman, Iram; Singaram, Veena

HEALTH SA GESONDHEID 2022;27(1):1-10

, <Blank> African Online Scientific Information System PTY LTD 2022

DOI: [10.4102/hsag.v27i0.1942](https://dx.doi.org/10.4102/hsag.v27i0.1942)

**Reasons for exclusion:** Wrong intervention

#687 - Rush 2018

Paving the Path to Mindfulness: Implementation of a Program to Reduce Stress and Burnout in Inpatient Psychiatric Nurses

Rush, Kristin Elizabeth

Paving The Path To Mindfulness: Implementation Of A Program To Reduce Stress & Burnout In Inpatient Psychiatric Nurses 2018;():1-1

University of North Carolina at Chapel Hill 2018

**Reasons for exclusion:** Wrong intervention

#4325 - LynnDobkin 2020

Physicians' Views on a Wellbeing Course Gifted to Them: A Qualitative Study X

Lynn Dobkin, P.; Velez, C.

Perm J Nov 2020;24():1-8

2020 Nov

DOI: [10.7812/tpp/19.228](https://dx.doi.org/10.7812/tpp/19.228)

**Reasons for exclusion:** Wrong intervention (discussed with a third researcher)

**Researcher who assessed the articles: Randi Karkov Knudsen and Connie Timmermann**

#9342 - Sharma 2020

A Qualitative Analysis Of Mindful Self Compassion to Improve Work Related Stress in Healthcare Professionals X

Sharma, Rita

ProQuest Dissertations and Theses 2020;(28093408):94

United States -- Illinois The Chicago School of Professional Psychology 2020

**Reasons for exclusion:** Wrong settting

#12058 - Munyoki 2022

Evaluation of a mindfulness-based stress reduction program to reduce stress, burnout, and insomnia for behavioral healthcare staff. X

Munyoki, Naumi

Dissertation Abstracts International: Section B: The Sciences and Engineering / 2022;83(11-B):No-Specified

US ProQuest Information & LearningUS 2022 /

Ref ID: 2022-70649-149

**Reasons for exclusion:** Wrong settting

#9447 - Adams 2018

Clinician Perspectives on Mindfulness-Based Interventions in Clinical Settings: A Qualitative Study X

Adams, Brooke Nicole

ProQuest Dissertations and Theses 2018;(13812831):165

United States -- Illinois The Chicago School of Professional Psychology 2018

**Reasons for exclusion:** Wrong settting

#12076 - Sos 2022

Comparison of mindfulness practices for effectiveness of stress and burnout reduction in healthcare staff. X

Sos, Tammy Elizabeth

Dissertation Abstracts International: Section B: The Sciences and Engineering / 2022;83(3-B):No-Specified

US ProQuest Information & LearningUS 2022 /

Ref ID: 2021-94599-254

**Reasons for exclusion:** Wrong setting

#643 - Haskins 2018

Mind over Matter: Enhancing Compassion Satisfaction in Oncology Nursing

Haskins, Jacob R.

Mind Over Matter: Enhancing Compassion Satisfaction In Oncology Nursing 2018;():1-1

University of North Carolina at Chapel Hill 2018

**Reasons for exclusion:** Wrong intervention

#934 - Wampole 2020

Exploring a social work lead mindfulness-based intervention to address burnout among inpatient psychiatric nurses: a pilot study X

Wampole, Donna M.; Bressi, Sara

Social Work in Health Care 2020;59(8):615-630

Oxfordshire, <Blank> Routledge 2020

DOI: [10.1080/00981389.2020.1827123](https://dx.doi.org/10.1080/00981389.2020.1827123)

**Reasons for exclusion:** Data based on survey

#218 - Sandvik 2011

Mindfulness - the aware nurse

Sandvik, Berit Andersen

Norwegian Journal of Clinical Nursing / Sykepleien Forskning 2011;(3):274-281

, <Blank> Sykepleien Forskning 2011

**Reasons for exclusion:** No qualitative data on factors affecting implementation of mindfulness in hospital settings

#4049 - Resnicoff 2018

Brief Mindfulness Meditation With Night Nursing Unit Staff: A Qualitative Study

Resnicoff, M.; Julliard, K.

Holist Nurs Pract Nov/Dec 2018;32(6):307-315

2018 Nov/Dec

DOI: [10.1097/hnp.0000000000000293](https://dx.doi.org/10.1097/hnp.0000000000000293)

**Reasons for exclusion:** No qualitative data on factors affecting implementation of mindfulness in hospital settings

#7896 - Drury 2014

Compassion satisfaction, compassion fatigue, anxiety, depression and stress in registered nurses in Australia: Phase 2 results

Drury, V.; Craigie, M.; Francis, K.; Aoun, S.; Hegney, D. G.

Journal of Nursing Management MAY 2014;22(4):519-531

2014 MAY

DOI: [10.1111/jonm.12168](https://dx.doi.org/10.1111/jonm.12168)

**Reasons for exclusion:** No qualitative data on factors affecting implementation of mindfulness in hospital settings

#4291 - Hazlett-Stevens 2020

Mindfulness-Based Stress Reduction for Health Care Staff: Expanding Holistic Nursing Paradigms to the Whole System

Hazlett-Stevens, H.

Holist Nurs Pract Sep-Oct 01 2020;34(5):301-305

2020 Sep-Oct 01

DOI: [10.1097/hnp.0000000000000404](https://dx.doi.org/10.1097/hnp.0000000000000404)

**Reasons for exclusion:** No qualitative data on factors affecting implementation of mindfulness in hospital settings

#13088 - Moorhead 2016

Becoming a more mindful practitioner: The effectiveness of a Mindfulness-based CBT course in meeting training needs for clinical staff in adult mental health Urgent Care services.

Moorhead, Cate; Winfield, Jill; Freeston, Mark H

the Cognitive Behaviour Therapist / 2016;9(Baer, R. A. (2003). Mindfulness training as a clinical intervention: a conceptual and empirical review. Clinical Psychology: Science and Practice 10, 125-143. 2003-03824-001. https://dx.doi.org/10.1093/clipsy/bpg015Bishop, S. R., Lau, M., Shapiro, S., Car):

United Kingdom Cambridge University PressUnited Kingdom 2016 /

DOI: [10.1017/S1754470X15000744](https://dx.doi.org/10.1017/S1754470X15000744) **·** Ref ID: 2016-06893-00

**Reasons for exclusion:** No qualitative data on factors affecting implementation of mindfulness in hospital settings

#13263 - Bohlinger 2014

Teaching self-compassion to decrease performance anxiety in clinicians.

Bohlinger, Anna I; Wahlig, Jeni L; Trudeau-Hern, Stephanie

Clinical supervision activities for increasing competence and self-awareness. / 2014;(Bowen, M. (1978). Family therapy in clinical practice. New York, NY: Jason Aronson.Burns, R. B. (1979). The self concept in theory, measurement, development and behavior. New York, NY: Longman.Cohn, M. A., & Fredrickson, B. L. (2010). In search of durable):61-65

Hoboken, NJ, US John Wiley & Sons, Inc.US 2014 /

Ref. ID: 2014-05687-010

**Reasons for exclusion:** No qualitative data on factors affecting implementation of mindfulness in hospital settings

| ID | # |
| --- | --- |
| Study no. |  |
| Title |  |
| Authors |  |
| Year |  |
| Country |  |
| Journal |  |
| Study design |  |

**Supplementary Material File 4:** Reviewer guide for extracting data

| **Methods** |  |  |
| --- | --- | --- |
| Aim of the study |  |  |
| Method for data collection |  |  |
| When was the data collected |  |  |
| Participants (number, profession) |  |  |
| Setting (where are the participant employed) |  |  |
| Type of analysis |  |  |
|  |  |  |
| **Type of intervention** |  |  |
| Description of type, duration, and delivery form, setting for the practice |  |  |
|  |  |  |
| **Data from results/discussion** |  |  |
| Overall themes (if any) |  |  |
|  |  |  |
| Results/Data/themes on facilitators for mindfulness implementation in hospitals (short description) |  |  |
|  |  |  |
| Results/Data/themes on inhibiting factors for mindfulness implementation in hospitals |  |  |

**Supplementary Material File 5:** The analysis process

| **Examples of quotations from articles** | **Codes** | **Preliminary themes** | **Analytic themes** |
| --- | --- | --- | --- |
| The challenges related to introducing programs and testing their effectiveness include enabling staff to participate in such a program, motivating ongoing engagement in mindfulness practice and supporting the integration of new ways of working into the workplace (Foureur et al., 2013). | Motivation | Buy-in | Buying in |
| This buy-in process occurred at every level of the department's hierarchy in a vertical and horizontal fashion. Leadership involved in the planning process pitched the value-add of mindfulness vertically, orienting leadership at all levels of the unit's organizational hierarchy to this new formal policy initiative during pre-existing meeting times (Byron et al., 2015).  Speak to the potential benefits that they can relate to (Weisbaum, 2021). | Pitched the perceived value |  |  |
| Misconceptions regarding the value of ESRT (Enhanced Stress Resilience Training) for surgeons, limited awareness of the evidence supporting MBI effectiveness, and perceived conflicts between well-being interventions and surgical ethos and identity (Lebares et al., 2020).  Some participants described becoming aware that they felt guilty when taking time for themselves, a requirement of the daily home practice of the MBSR program (Irving et al., 2014).  Creative strategies need to be developed for further/future workshop participation for nurses and midwives whose self-care needs are often postponed or neglected, as the needs of others become their priority (Foureur et al., 2013). | Attitudes, beliefs, values |  |  |
| Staff described the fact that participation was optional and offered as an invitation rather than an expectation facilitated their participation (Byron et al., 2015). | Optional – not a demand |  |  |
| Factors internal to the individual, including fatigue, thinking, and intense or negative emotions, could also influence the success of mindfulness practice (Lyddy et al., 2016).  Dealing with difficult emotions “This weekend, I found it extremely difficult get back to practice. After the retreat I had many negative feelings. Glad I pushed myself to practice (Cohen-Katz et al., 2005). | Personals challenges | Physical or emotional challenges |  |
|  |  |  |  |
| For each participant, negotiating time off the ward to attend the one-day workshop to which they were allocated proved to be challenging as rosters were often changed at short notice to accommodate the needs of stafﬁng and workloads. Participants ultimately attended whichever workshop they could ﬁt into their roster (Foureur et al., 2013).  Suspending busyness could be challenging and frustrating (Hunter et al., 2018).  One participant pointed out that practicing mindfulness exercises was sometimes hard and difficult to incorporate into a busy workday, with the risk of giving up (Knudsen et al., 2021). | Time constraints | Time and space | Allocating time and space |
| External barriers included busy schedules and home environments wherein it was difficult to find time alone. Also, a work  environment that required acting in a manner identified as “counter to mindfulness” such as constant rushing from patient to the next and requisite multitasking were named as impediments (Irving et al., 2014).  The barriers for an 8-week MBCT intervention to adherence as identified by the focus groups were related to the required face-to-face sessions (Mealer et al., 2017). | Space for practicing |  |  |
| At the organizational level, support from hospital administration is needed so that nurses caring for HIV infected patients may attend MLWS interventions (Pan et al., 2019).  The process began with leadership’s buy-in effort, pitching mindfulness practice to employees during pre-existing meeting times as a non-mandatory training option to improve self-care. By design, classes included direct care providers (social workers, nurses, and psychiatrists), as well as program and unit leadership (Byron et al., 2015).  We operationalized Allowance as the presence or absence of respect, retribution or recrimination for intervention participants, recognizing the potency of leadership in inﬂuencing prevailing attitudes (Lebares et al., 2020). | Leadership support |  |  |
| The interviewed participants provided useful insight into the feasibility of the program, specific to the timing of the sessions, as well as the integration of technology to deliver session information. The use of videoconferencing to stream the sessions provided participants with attendance flexibility (Muir and Keim-Malpass, 2020). | Online technology |  |  |
|  |  |  |  |
| Notably, the majority of the interviewees reported that they practiced informal mindfulness techniques, whereas formal practice was less frequent. Furthermore, the resident physicians stated that they were able to integrate those informal practices into their work life, such as mindful walking along the clinic corridor, pausing for a moment before continuing their work, mindful eating, or taking a few conscious breaths while disinfecting their hands (Aeschbach et al., 2021).  Informal rather than formal practice It seemed that the majority of the participants found informal applications of mindfulness practice more useful and realistic to implement than the formal techniques. Examples given of this include focusing on one’s breathing and pausing to become aware of external and internal factors (Negus and Grobler, 2021). | Content – informal practice | Informal practice | Keeping it going |
| Attending a mindfulness course with a group of previously known or unknown colleagues initiated a process of group bonding which participants found incredibly supportive and beneficial (Hunter et al., 2018).  Almost all resident physicians reported that they had experienced it as helpful and supportive to attend the MBP with fellow resident physicians (Aeschbach et al., 2021). | Group training | Group training |  |
| The commitment of the mindfulness expert and nurse manager was also found to be characteristic of the diffusion of innovations. They acted as early adopters serving to diffuse mindfulness throughout the units via formal and informal communication and influence (Byron et al., 2015). | Experts/Local champions/networks | Support for sustainability |  |
| When asked what they would like to change in the program, the participants often mentioned a need for post-training follow-up and reminders: “So what I would like is to have something after […] a reminder” (Interviews 5, 6, 7, and 10). One person also reported that the program should offer more intersessional follow-up to encourage the sharing of feelings and changes that they were experiencing (Brun et al., 2023).  Many indicated a wish for some sort of follow-up with other health care professionals (Irving et al., 2014). | Follow-up |  |  |

**Supplementary Material File 6**

| **Review findings** | **Number of contributing studies** | **References** |
| --- | --- | --- |
| **Theme 1: Buying in** |  |  |
| *Inhibiting factors* |  |  |
| Prejudices and misconceptions about mindfulness | 3 | (Aeschbach et al., 2021, Lebares et al., 2020, Weisbaum, 2021) |
| Down-prioritizing and/or feeling guilty of self-care | 6 | (Cohen-Katz et al., 2005, Hunter et al., 2018, Irving et al., 2014, Lebares et al., 2020, Muir and Keim-Malpass, 2020, Negus and Grobler, 2021) |
| Concerns about showing vulnerability in front of peers | 2 | (Nissim et al., 2019, Slatyer et al., 2018). |
| Physical and emotional challenges with practicing mindfulness | 7 | (Cohen-Katz et al., 2005, Foureur et al., 2013, Hunter et al., 2018, Irving et al., 2014, Lyddy et al., 2016, Negus and Grobler, 2021, Weisbaum, 2021) |
| *Facilitating factors* |  |  |
| Emphasize the value of mindfulness training to the work specific practice, including past participants experience and the evidence base of the effectiveness of mindfulness-based interventions | 4 | (Byron et al., 2015, Lebares et al., 2020, Mealer et al., 2017, Weisbaum, 2021) |
| Associate the training with better patient care | 2 | (Lebares et al., 2020, Weisbaum, 2021) |
| Attending to inter-personal relationships in the local context when composing the groups | 3 | (Nissim et al., 2019, Slatyer et al., 2018, Weisbaum, 2021) |
| Creating a safe space, preparing for potential discomfort, making participation optional, and leaving room to different levels of engagement | 4 | (Byron et al., 2015, Irving et al., 2014, Nissim et al., 2019, Weisbaum, 2021) |
| **Theme 2: Allocating time and space** |  |  |
| *Inhibiting factors* |  |  |
| Lack of time | 9 | (Aeschbach et al., 2021, Byron et al., 2015, Foureur et al., 2013, Hunter et al., 2018, Irving et al., 2014, Knudsen et al., 2021, Lyddy et al., 2016, Negus and Grobler, 2021, Pan et al., 2019). |
| Space for engaging in mindfulness practice | 3 | (Byron et al., 2015, Lyddy et al., 2016, Mealer et al., 2017) |
| *Facilitating factors* |  |  |
| Leadership support in terms of securing protected time during working hours to attend mindfulness training and secure personal coverage | 3 | (Byron et al., 2015, Lebares et al., 2020, Nissim et al., 2019) |
| Offering mindfulness training in-house but with the possibility to participate online if physical attendance is an obstacle for participating | 2 | (Mealer et al., 2017, Muir and Keim-Malpass, 2020) |
| **Theme 3: Keeping it going** |  |  |
| *Inhibiting factors* |  | *Facilitating factors* |
| Gaining knowledge from a book | 1 | (Aeschbach et al., 2021). |
| Changing habits and sustaining the benefits of mindfulness training | 7 | (Aeschbach et al., 2021, Cohen-Katz et al., 2005, Foureur et al., 2013, Hunter et al., 2018, Knudsen et al., 2021, Lyddy et al., 2016, Nissim et al., 2019) |
| *Facilitating factors* |  |  |
| Getting firsthand experiences with mindfulness including learning informal mindfulness practices, which can be integrated directly in practice | 14 | (Aeschbach et al., 2021, Brun et al., 2023, Foureur et al., 2013, Hunter et al., 2018, Irving et al., 2014, Knudsen et al., 2021, Lyddy et al., 2016, Mealer et al., 2017, Minichiello et al., 2020, Negus and Grobler, 2021, Nissim et al., 2019, Pan et al., 2019, Slatyer et al., 2018, Weisbaum, 2021). |
| Follow-up training | 9 | (Brun et al., 2023, Cohen-Katz et al., 2005, Foureur et al., 2013, Hunter et al., 2018, Irving et al., 2014, Knudsen et al., 2021, Nissim et al., 2019, Pan et al., 2019, Slatyer et al., 2018). |
| Offering mindfulness training in a group. The group training is supportive, helps with overcoming challenges and applying it to clinical practice | 10 | (Aeschbach et al., 2021, Brun et al., 2023, Byron et al., 2015, Cohen-Katz et al., 2005, Irving et al., 2014, Knudsen et al., 2021, Lyddy et al., 2016, Muir and Keim-Malpass, 2020, Slatyer et al., 2018, Weisbaum, 2021). |
| Identifying committed local champions serving to diffuse mindfulness through the implementation process | 1 | (Byron et al., 2015) |

Aeschbach, V.M., Fendel, J.C., Schmidt, S., Göritz, A.S., 2021. A tailored mindfulness-based program for resident physicians: A qualitative study. Complementary Therapies in Clinical Practice 43, 101333. 10.1016/j.ctcp.2021.101333

Brun, C., Akinyemi, A., Houtin, L., Mizzi, C., Cardoso, T., Isnard Bagnis, C., 2023. Mindfulness and compassion training for health professionals: A qualitative study. Frontiers in Psychology 13, 1113453. 10.3389/fpsyg.2022.1113453

Byron, G., Ziedonis, D.M., McGrath, C., Frazier, J.A., deTorrijos, F., Fulwiler, C., 2015. Implementation of Mindfulness Training for Mental Health Staff: Organizational Context and Stakeholder Perspectives. Mindfulness (N Y) 6 (4), 861-872. 10.1007/s12671-014-0330-2

Cohen-Katz, J., Wiley, S., Capuano, T., Baker, D.M., Deitrick, L., Shapiro, S., 2005. The effects of mindfulness-based stress reduction on nurse stress and burnout: a qualitative and quantitative study, part III. Holistic Nursing Practice 19 (2), 78-86. 10.1097/00004650-200503000-00009

Cohen-Katz, J., Wiley, S., Capuano, T., Baker, D.M., Deitrick, L., Shapiro, S., 2005. The effects of mindfulness-based stress reduction on nurse stress and burnout: a qualitative and quantitative study, part III. Holist Nurs Pract 19 (2), 78-86. 10.1097/00004650-200503000-00009

Foureur, M., Besley, K., Burton, G., Yu, N., Crisp, J., 2013. Enhancing the resilience of nurses and midwives: Pilot of a mindfulnessbased program for increased health, sense of coherence and decreased depression, anxiety and stress. Contemporary Nurse 45 (1), 114-125. 10.5172/conu.2013.45.1.114

Hunter, L., Snow, S., Warriner, S., 2018. Being there and reconnecting: Midwives' perceptions of the impact of Mindfulness training on their practice. Journal of Clinical Nursing 27 (5-6), 1227-1238. 10.1111/jocn.14169

Irving, J.A., Park-Saltzman, J., Fitzpatrick, M., Dobkin, P.L., Chen, A., Hutchinson, T., 2014. Experiences of health care professionals enrolled in mindfulness-based medical practice: A grounded theory model. Mindfulness 5 (1), 60-71. <https://dx.doi.org/10.1007/s12671-012-0147-9>

Knudsen, R.K., Gregersen, T., Ammentorp, J., Tousig, C.G., Timmermann, C., 2021. Healthcare professionals’ experiences of using mindfulness training in a cardiology department – a qualitative study. Scandinavian Journal of Caring Sciences 35 (3), 892-900. 10.1111/scs.12906

Lebares, C.C., Guvva, E.V., Desai, A., Herschberger, A., Ascher, N.L., Harris, H.W., O'Sullivan, P., 2020. Key factors for implementing mindfulness-based burnout interventions in surgery. AMERICAN JOURNAL OF SURGERY 219 (2), 328-334. 10.1016/j.amjsurg.2019.10.025

Lyddy, C.J., Schachter, Y., Reyer, A., Julliard, K., 2016. Transfer of mindfulness training to the work setting: A qualitative study in a health care system. Journal of Continuing Education in the Health Professions 36 (4), 240-248. 10.1097/CEH.0000000000000120

Mealer, M., Hodapp, R., Conrad, D., Dimidjian, S., Rothbaum, B.O., Moss, M., 2017. Designing a resilience program for critical care nurses. AACN Advanced Critical Care 28 (4), 359-365. 10.4037/aacnacc2017252

Minichiello, V., Hayer, S., Gillespie, B., Goss, M., Barrett, B., 2020. Developing a mindfulness skills-based training program for resident physicians. Family Medicine 52 (1), 48-52. 10.22454/FamMed.2020.461348

Muir, K.J., Keim-Malpass, J., 2020. The Emergency Resiliency Initiative: A Pilot Mindfulness Intervention Program. Journal of Holistic Nursing 38 (2), 205-220. 10.1177/0898010119874971

Negus, N.H., Grobler, G., 2021. How can a 6-week training course shape mental healthcare professionals’ understanding of mindfulness? Experiences at weskoppies psychiatric hospital. South African Journal of Psychiatry 27, 1489. 10.4102/sajpsychiatry.v27i0.1489

Nissim, R., Malfitano, C., Coleman, M., Rodin, G., Elliott, M., 2019. A Qualitative Study of a Compassion, Presence, and Resilience Training for Oncology Interprofessional Teams. Journal of Holistic Nursing 37 (1), 30-44. 10.1177/0898010118765016

Pan, C., Wang, H., Chen, M., Cai, Y., Xiao, C., Tang, Q., Koniak-Griffin, D., 2019. Mindfulness-based intervention for nurses in AIDS care in China: A pilot study. Neuropsychiatric Disease and Treatment 15, 3131-3141. 10.2147/NDT.S223036

Slatyer, S., Craigie, M., Rees, C., Davis, S., Dolan, T., Hegney, D., 2018. Nurse Experience of Participation in a Mindfulness-Based Self-Care and Resiliency Intervention. Mindfulness 9 (2), 610-617. 10.1007/s12671-017-0802-2

Weisbaum, E.A., 2021. Applied Mindfulness for Physicians: A Prospective Qualitative Study. University of Toronto (Canada), Canada -- Ontario, CA, pp. 1-419.
